# Metabolism and Bioavailability of Olive Bioactive Constituents Based on In Vitro, In Vivo and Human Studies

**DOI:** 10.3390/nu14183773

**Published:** 2022-09-13

**Authors:** Theodora Nikou, Maria Eleni Sakavitsi, Evangelos Kalampokis, Maria Halabalaki

**Affiliations:** Division of Pharmacognosy and Natural Products Chemistry, Department of Pharmacy, National and Kapodistrian University of Athens, 15771 Athens, Greece

**Keywords:** metabolism, ADMET properties, in vitro assays, in vivo, human studies, hydroxytyrosol, tyrosol, oleuropein, oleocanthal, oleacein

## Abstract

Consumption of olive products has been established as a health-promoting dietary pattern due to their high content in compounds with eminent pharmacological properties and well-described bioactivities. However, their metabolism has not yet been fully described. The present critical review aimed to gather all scientific data of the past two decades regarding the absorption and metabolism of the foremost olive compounds, specifically of the phenylalcohols hydroxytyrosol (HTyr) and tyrosol (Tyr) and the secoiridoids oleacein (Olea), oleocanthal (Oleo) and oleuropein (Oleu). A meticulous record of the in vitro assays and in vivo (animals and humans) studies of the characteristic olive compounds was cited, and a critical discussion on their bioavailability and metabolism was performed taking into account data from their gut microbial metabolism. The existing critical review summarizes the existing knowledge regarding the bioavailability and metabolism of olive-characteristic phenylalchohols and secoiridoids and spotlights the lack of data for specific chemical groups and compounds. Critical observations and conclusions were derived from correlating structure with bioavailability data, while results from in vitro, animal and human studies were compared and discussed, giving significant insight to the future design of research approaches for the total bioavailability and metabolism exploration thereof.

## 1. Introduction

The consumption of olive tree (*Olea europaea* L.—Oleaceae) products, such as olive oil (OO) and table olives, key components of the Mediterranean diet (MD), is now synonymous with a healthy way of eating and living. The positive health impact of olive products is attributed to their exceptional chemical composition [1]. For instance, OO is characterized by a unique mixture of esters of unsaturated fatty acids, such as oleic, linoleic and linolenic acids. Furthermore, apart from the lipophilic fraction of OO, in recent years, a different group of constituents has been placed in the scientific spotlight due to its members’ significant biological activity—the so-called “olive oil polyphenols or biophenols (OBs)” [2]. It regards relatively polar compounds, which are found in small concentrations and belong to diverse chemical classes (e.g., phenylalcohols, phenolic acids, secoiridoids, flavonoids, terpenoids, lignans, hydroxy-isocromans, etc.) [3]. Amongst them, the phenylalcohols tyrosol (Tyr) and hydroxytyrosol (HTyr)—together with the secoiridoids oleacein (Olea) and oleocanthal (Oleo)—are so far the most characteristic and studied OBs for their pharmacological properties [1,4]. Moreover, the secoiridoid glucoside oleuropein (Oleu), which is abundant in olive drupes and leaves and found only in traces in OO, is considered to be a significant bioactive, since a plethora of studies for its benefits to human health are available [5,6,7,8,9].

**Figure 1:**
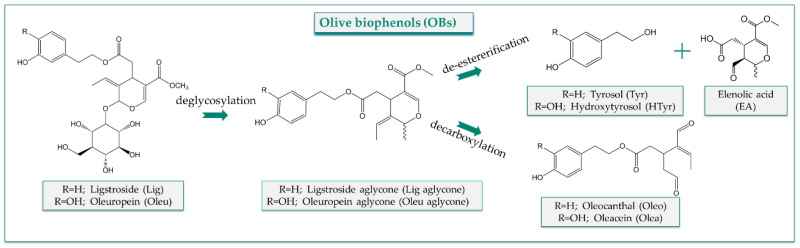
Chemical structures and formation of oleuropein (Oleu) and ligstroside (Lig) aglycones, oleocanthal (Oleo), oleacein (Olea), hydroxytyrosol (HTyr) and tyrosol (Tyr).

The OBs, acting as extra nutrients, seem to be responsible for diverse health-promoting and disease-preventing properties and are genuinely associated with the beneficial health effects of the MD [10]. HTyr and Tyr, apart from olive products, are also present in other species of Oleaceae and in red wine, and they are also endogenously formed in humans from dopamine and tyramine metabolism, respectively [11,12]. The secoiridoids (Olea, Oleo, Oleu) penetrate to humans exclusively through consumption of olive products, expressing their protective and/or therapeutic effects. From a chemical point of view, HTyr is a hydroxylated derivative of Tyr, and their esters with elenolic acid (EA) form Oleu and Lig aglycones, respectively. The glycosylated derivatives of the later lead to Oleu and Lig, whereas the loss of carboxymethyl moiety of the aglycone forms with the simultaneous opening of the secoiridoid ring leads to Olea and Oleo (Figure 1).

Numerous in vitro, in vivo and clinical studies have been performed over the last decade to determine the beneficial effects of OBs to human health [13,14,15]. Specifically, research on OBs has shown that there is a strong association of OBs with the prevention of certain pathologies in humans, placing them at the center of scientific interest. Research evidence has shown that OBs contribute to the improvement of human lipid profile and enhance high-density lipoprotein function [16], lower systolic pressure and prevent hypertension [17], regulate blood glucose [18] and reduce pro-inflammatory state [19] and oxidative stress [20]. These are just few of the studied beneficial effects of OBs to human health associated with several chronic and age-related disorders such as cancer, cardiovascular and neurodegenerative diseases [21,22,23]. Additionally, OBs have been found to protect against several metabolic disorders, such as insulin resistance [24] and obesity [25]. Based on experimental observations, OBs benefits are closely related to their chemical structure and their protective role against several diseases is attributed to their strong antioxidant and anti-inflammatory activity. They have been found to act as efficient free radical scavengers and metal ion chelators, counteracting the cytotoxic effects of oxidative stress in the organism [4,7,26]. In conjunction with the direct scavenging of reactive species, certain reports have demonstrated that the modulation of gene expression plays a crucial role in the antioxidant and anti-inflammatory properties of OBs [4]. Their anti-inflammatory properties are achieved by down-regulating inflammatory mediators via transcriptional or post-transcriptional mechanisms and by modulating the activation of kinases involved in the onset of inflammatory process at different levels [27,28]. Since oxidative stress pathways and inflammation are linked to numerous pathologies (neurodegenerative, cardiovascular and digestive disorders and cancer) the antioxidant and anti-inflammatory synergistic effects of OBs have been associated to the bioactivity of extra virgin OO against age-related and chronic diseases [1,4].

At this point, it must be noted that pivotal role for the demonstration of a biological and/or pharmacological function for natural products—and so for food bioactive compounds, such as OBs—is their bioavailability in humans [29]. Assessment of bioaccessibility and/or bioavailability can be performed in vitro by using special cell line studies or in vivo with animal models and human biological fluids. The meaning of bioavailability describes a nutrient or food bioactive which is efficiently digested, absorbed and become available to provide its beneficial effect to the organism, while bioaccessibility is the amount of each food bioactive that is efficiently released from the respective food matrix, possibly permeates into the gastrointestinal (GI) tract and if so, it is eventually absorbed from the organism [30]. Thus, bioavailability includes bioaccessibility of orally administered food bioactives. Additionally, pharmacokinetics (PK) are incorporated into determining the fate of a compound in in vivo systems through the investigation of compound absorption, distribution, metabolism and excretion (and toxicity) or ADME(T) studies [31]. The key element in these studies is the investigation of metabolism, the process by which xenobiotics (in our study’s case, OBs as pure chemical entities, food constituents, enriched extracts or supplements) and endogenous metabolites are converted enzymatically into more hydrophilic and easily excreted compounds [32]. Formed or biotransformed metabolites of the parent molecule might possess similar or different activity or even potential toxicity [32]. Such studies are fundamental in a drug’s development process. However, they are sporadic in the area of food bioactives and rather rare in the area of natural products [33].

To that end, it is vital to understand the biotransformation of a parent compound into other analogues or derivatives that could possibly act as accomplices and/or competitors in the demonstration of a biological function; thus, acquaintance with the metabolism of a food bioactive compound is highly important [34]. Metabolism takes place in various tissues, of which liver and intestine are the main sites for orally administered compounds. Metabolism transformations can be divided into phase I and phase II reactions. Oxidation, hydrolysis and reduction are common phase I reactions mainly occurring in the stomach, liver and gut wall. Phase II reactions, also called conjugation reactions, occur mostly in the liver, but also in the small intestine and involve the addition of glucuronic, sulfate, glutathionyl, acetyl and methyl moieties on the molecule, catalyzed by the respective enzymes [35]. A compound often goes through phase I before phase II transformation. The phase III detoxification system must be mentioned as a part of a compound’s metabolism process since it has the important task of eliminating toxic metabolites from cells [36]. Finally, human gut microbiota makes key contributions to metabolism, transforming hundreds of food bioactives into metabolites with altered activity, toxicity and lifetime within the body [37].

Several scientific approaches have been proposed for the investigation of the metabolism of food bioactives, though is still remains mostly unexplored. This is especially true for OBs; despite the plethora of bibliographical data concerning their biological activity in vitro, in vivo and in human trials, the studies focusing on their bioavailability and metabolism are quite restricted. Routinely, in vitro assays are used to establish the metabolic profile of a moiety, incorporating microsomes, supersomes, cytosol, S-9 fraction and cell-based models (primary hepatocytes, liver slices and perfused liver), which contain metabolism enzymes or are tested for evaluation of compounds’ intestinal permeability [35]. Additionally, complementary in silico models are used to assist in vitro screenings and the prediction of the metabolism catalyzed by the respective enzymes [38]. In vitro bioaccessibility experiments are usually employed as an important first step in studying the influence of gastrointestinal digestion and food matrices on the bioavailability of compounds. The succeeding step is in vivo studies conducted to evaluate PK parameters. In vivo PK studies are performed with animals, such as mice and rats, to generate PK data and determine compounds’ clearance, bioavailability, exposure, half-life, distribution volume and toxicity [39,40]. As expected, the final go/no-go decision will be made after studies in humans. For food bioactives, such as OO and OBs, usually dietary interventions are designed monitoring C_max_ and T_max_ in human biological fluids (blood plasma) and excretions (urine, feces), which are investigated to detect the existence of possible metabolic derivatives of the administered compounds [41].

However, exploration of the metabolism of food bioactives is hindered by the existence of many complications. The use of low dosages, the complexity of the tested active entity in cases of extract supplementation (e.g., OBs) or foodstuffs (enriched with OBs OO) as well as unavailability of reference standards are regarded as critical complications in the field of natural product bioavailability and metabolism studies [40]. Therefore, the analytical platform which prescribes the limits of detection and sensitivity is of great importance as well as the preparation of samples prior analysis for the recovery of target compound(s) is used. For instance, OBs bind strongly to plasma proteins, hampering recovery prior to analysis, detection efficiency and the establishment of appropriate quantitation protocols [42]. For this purpose, alternative scientific approaches and more sophisticated methodologies are gradually incorporated to address these constraints. For example, metabolomics, which propose a holistic data mining and interpretation, have recently offered new opportunities to investigate metabolism patterns and pathways [43].

Additionally, the large interindividual variability affecting ADMET parameters, such as age, sex, dietary habits, microbiome composition, genetic variation, drug exposure and many other factors, complicate food bioactive evaluation further. Regarding OBs, few studies have reported individual data on their ADMET properties and on determinants such as the aforementioned factors [36,44]. Additionally, human gut microbiota complicates more the monitoring of OBs’ metabolic fate. OBs known to survive phase I and II metabolism finally undergo colonic metabolism by human gut microbiota. It has long been speculated by data in the literature that the microbial population of the human gut is a major contributor to the overall metabolism of not only orally submitted bioactive compounds but also of phase I and II metabolites that have been excreted back into the intestine via enterohepatic circulation [45,46]. In addition, the normal levels of endogenously produced metabolic derivatives in human biological fluids, such as the phenylalcohols HTyr and Tyr, should be taken into consideration in the design of such studies, and their PK parameters should be more carefully evaluated to reach reasonable conclusions.

More specifically, the information concerning the bioavailability of most OBs is limited despite the intensive research that has been devoted the past two decades to the investigation of their biological properties. This fact is reflected in the number of review papers published from 2002 to 2022 [29,47,48,49,50,51]. Regarding OBs’ metabolism, sporadic reviews have been published focusing either on specific compounds [12] or specific approaches [52], or—in most cases due to the high variety of chemical categories and extremely high number of different compounds included in each olive product—they are investigated and discussed separately [34,50]. Additionally, although over the past decade, the studies on colonic metabolism and modulation of gut microbiota by dietary compounds have gained scientific attention, bibliographical data that cover the colonic biotransformation of OBs are limited so far [52]. Finally, the data regarding humans are limited to the study of metabolism after HTyr or OO supplementation [53].

Therefore, the study of food bioactives bioavailability and metabolism, specifically of OBs, requires significant research effort and multilateral experimental approaches to encompass the open questions and to reach solid conclusions. Hence, to address the bioavailability of OBs, in vitro, in vivo and human studies on the absorption, transportation, metabolism and excretion of OBs have been reviewed. In the following pages, this review focuses on gathering experimental protocols and pre-clinical studies, while intervention-based clinical trials are discussed as well. Additionally, attention is given to their metabolism from gut microbiota to enlighten a new research field of OBs metabolism. Results were categorized and presented according to the chemical group of compounds, especially for phenylalcohols and secoiridoids, which represent the most characteristic chemical groups of olive products. The gathered data were systematically discussed, correlating phenylalcohol and secoiridoid structures to compounds’ metabolic fate to reveal their basic metabolic differences. Finally, the present critical work is a conclusive literature review discussing and emphasize all grey areas in the bioavailability and metabolism of OBs.

## 2. Materials and Methods

A systematic literature research of Scopus, PubMed, Web of Science, CrossRef and ScienceDirect databases was carried out. The original articles investigating the bioavailability of main olive phenolic compounds were identified using the following search keywords: hydroxytyrosol (or 3,4-Dihydroxyphenylethanol); tyrosol (or 2-(4-Hydroxyphenyl)ethanol); oleuropein; oleocanthal (or decarboxymethylligstroside aglycone or 2-(4-hydroxyphenyl)ethyl (E,3S)-4-formyl-3-(2-oxoethyl)hex-4-enoate); oleacein (or decarboxymethyloleuropein aglycone or 2-(3,4-dihydroxyphenyl)ethyl (4Z)-4-formyl-3-(2-oxoethyl)hex-4-enoate); olive oil phenols; olive polyphenols AND in vitro assays/in vivo/ADMET/Clinical trials OR bioavailability/bioaccessibility/absorption/distribution/metabolism/excretion covering the 2010–2022 time span. The literature was further manually selected according to relevance to the topic. The focus was on the new insights regarding bioavailability of the aforementioned biophenols as well as on OO in general. However, due to the limited data found for the bioavailability and metabolism of the studied compounds, the time span was broadened to cover important literature data before 2010, which was carefully chosen and introduced when needed based on its relevance and the importance of the findings. For sorting biographical results, inclusion and exclusion criteria were incorporated. Articles, or at least the abstract, must have been written in English or Spanish, while no restrictions were taken under consideration for the article sample size. Conference abstracts, reviews meta-analyses, case reports, ecological studies, and letters to the editor were excluded. Furthermore, Mendeley was used as archiving software and reference manager. Table 1 summarizes the inclusion and exclusion criteria of the review.

## 3. Results

### 3.1. Bibliography Overview

The literature search provided 211 articles. Among these, a total of 127 articles were finally included after applying the mentioned criteria, and 24 articles were additionally introduced to cover important literature data regarding the bioavailability and metabolism of olive-characteristic constituents before 2010.

**Figure 2:**
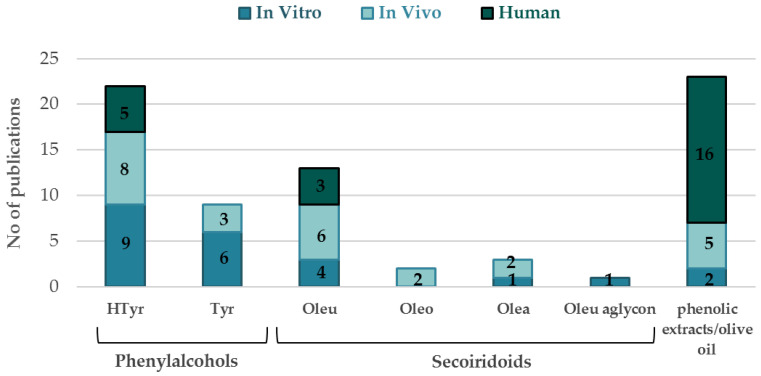
Bar chart of the number of research articles for the ADMET properties of olive-characteristic compounds categorized in in vitro, animal and human studies. In each bar, the respective number of publications is illustrated. HTyr: hydroxytyrosol, Tyr: tyrosol, Oleu: oleuropein, Oleo: oleocanthal, Olea: oleacein, Oleu aglycone: oleuropein aglycone, EOO: enriched olive oil. Time span 2000–2020.

In Figure 2, the bar chart visualizes the bibliographical data dedicated to the selected olive compounds regarding their ADMET properties, divided into three main categories of preclinical (in vitro, in vivo) and clinical (human) studies. Focus was given on research articles, the interpretation of their results and their final outcomes. In total, 31 research articles were found for phenylalcoholol metabolism, 19 for secoiridoids (Oleu, Oleu aglycone, Oleo and Olea) and 23 for olive extracts. The most-studied OBs were HTyr for phenylalcohols and Oleu for secoiridoids, and consequently, HTyr, Oleu and enriched OOs covered the majority of scientific literature presented in the current review. On the other hand, Tyr, Oleu aglycone, Olea and Oleo lack preclinical and clinical studies despite exhibiting significant biological activities. Additionally, review articles and book chapters were thoroughly studied to gather as much as possible of the existing knowledge around the metabolism, the metabolism of natural products and OBs and the current trends of metabolism studies to obtain a general overview of the subject as well as to uncover uncertainties or gaps in the existing literature. Therefore, an extended discussion on the data so far and a critical view will be provided, aiming to clarify and understand the fate of olive-characteristic compounds after their consumption as foodstuffs or as pure compounds in in vitro models and animal and human studies.

### 3.2. ADMET of Hydroxytyrosol (HTyr) and Tyrosol (Tyr)

OO phenylalcohols are small compounds regarding their molecular mass, yet show a plethora of health-promoting effects implicated in several biological mechanisms. Chemically, they belong to the phenylethylalcohols group, with HTyr being a hydroxylated on the aromatic ring derivative of Tyr [53]. Currently, HTyr is considered one of the most potent natural antioxidants [54].

HTyr and Tyr, the most well-studied phenylalcohols of olive products, have drawn research attention with their significant bioactivity profile—as pure compounds or as part of enriched extracts and OO—in in vitro and in vivo assays as well as in human interventions. At this point, it must be underlined that HTyr, together with its derivatives, is mainly responsible for a health claim regarding OO released by EFSA in 2011 related to a protective effect against low-density lipoproteins (LDL) oxidation above a certain concentration level [55]. Consequently, since then, several studies have been published regarding HTyr and its derivatives beneficial effects as well as its metabolic fate in biological systems. However, HTyr and Tyr are also produced endogenously in humans through tyramine and dopamine metabolism pathways. This information has raised additional questions on the bioavailability, pharmacokinetics and pharmacodynamics of these compounds regarding whether or not their entrance into the human system through food acts complementary or independently from those endogenously produced [29]. Metabolism and PK studies of phenylalcohols could enlighten the aforementioned question. Tables S1 and S2 summarize the existing bibliographical data regarding their bioavailability and metabolism investigation through in vitro assays and in vivo animal studies, discussed in detail in the next paragraphs.

#### 3.2.1. In Vitro Bioavailabilty and Metabolism Assays of Hydroxytyrosol (Htyr)

As mentioned on introduction, the effects of food bioactives, and therefore OBs, depend on their capacity to be absorbed and metabolized in the GI tract. In other words, to be bioaccessible. A detailed investigation of availability under GI conditions together with the study of transport and metabolism in Caco-2 cell monolayers and further hepatic metabolism by HepG2 cells is the basic line of research that has been reported for metabolism investigation of HTyr with in vitro models.

In 2000, a pioneering work by Manna et al. [56] using differentiated Caco-2 cell monolayers as a model system examined for the first time the mechanisms of HTyr intestinal permeation. More specifically, the authors investigated the kinetics of ^14^C-labeled HTyr intestinal transport and metabolism in Caco-2 cells. The study outcome supported that HTyr is absorbed via a passive diffusion mechanism bidirectionally and in a dose-dependent manner. Homovanillic alcohol (4-hydroxy-3-methoxy phenylethanol, HVAlc), a methylated derivative of HTyr, was also detected as a metabolic derivative. A second attempt using Caco-2 cell monolayers and segments of rat jejunum and ileum was performed by Corona et al. in 2006 [57]. HTyr was found to be transferred across the cell monolayers and segments of rat intestine and subjected to phase I and II biotransformation reactions. Like the previous research, HVAlc was detected as a metabolic derivative along with glutathionyl-HTyr.

In 2010, Soler et al. [58] employed a two-compartment trans well system containing human enterocytes (differentiated Caco-2/TC7) using monolayers, which simulate the small intestinal barrier. This study noticed a limited intestinal metabolism of HTyr. Its methylated derivatives were proposed as major metabolites, and for the first time, sulfated metabolites were annotated. The same year, Pereira. et.al. [59], using the same model, evaluated the intestinal permeation and metabolism of a series of ether derivatives of HTyr with different alkyl chain lengths (methyl, ethyl, propyl and butyl). Based on the authors’ observations, the rate of metabolism increased according to the lipophilicity of the derivatives (butyl > propyl > ethyl > methyl), indicating a possible better absorption rate of the more lipophilic compounds in comparison to the parent molecule. Glucuronides and methylated metabolites of HTyr alkyl ethers were detected in both the apical and basolateral compartments, but a significant portion (up to 80%) of the transported material was not metabolized.

In 2011, Mateos et al. [60], drew a similar conclusion on the transport and metabolism of HTyr. They found that the acetylated HTyr, as a more lipophilic compound in comparison to the parental form, was better absorbed and served to delivery enhancement of HTyr to enterocytes for subsequent metabolism and basolateral efflux. Additionally, in recent decades, the formulation of compounds/final products has gained extensive research attention as it has been found to contribute to delaying or preventing oxidation phenomena in the final products [61]. Following this concept, three years later—in 2014—a first approach to evaluate possible effects of the co-occurring compounds of OO on HTyr absorption was evaluated. An in vitro digestion model was used, and the authors concluded that the bioaccessibility of HTyr was enhanced when the simulated medium was combined with extracts of herbs, such as thyme. After the in vitro digestion, the digested extracts (considered the bioaccessible fraction) were subjected to a bioavailability study with Caco-2 cells. Authors found that similar proportions of metabolized HTyr were detected after the extract exposure, which indicated HTyr was not affected by the presence of thyme bioactives during the efflux across the epithelial cells. HTyr was almost completely converted into HTyr sulfate (HTyr-sulf), HTyr acetate sulfate and HVAlc sulfate (HVAlc-sulf), with most appearing on the basolateral side, which indicates extensive sulfation and methyl-sulfation during the first-pass metabolism in the epithelial cells [62].

The latest research on bioaccessibility and absorption of HTyr was performed to evaluate the simulated medium, either a food matrix or an aqueous solution of *β*-cyclodextrin, leading to the result that food matrix does alter in vitro HTyr bioavailability, whereas *β*-cyclodextrin does not [63,64]. In another approach, biocompatible water-in-oil (W/O) microemulsions were developed as hosts for HTyr and subsequently examined for their absorption profile. The absorption of HTyr in solution was compared with the encapsulated one in vitro using a co-culture model (Caco-2/TC7 and HT29-MTX cell lines). Results suggested that the higher the surfactants’ concentration in the system, the lower the HTyr concentration that penetrated the constructed epithelium, indicating the involvement of the amphiphiles in its absorption and its entrapment in the mucus layer [65]. Additionally, the investigation of the potential hepatic transformation of HTyr, HepG2 cells are used and incubated with HTyr. Extensive uptake and metabolism of HTyr has been observed, while mainly glucuronide and methyl conjugates have been proposed as HTyr metabolites [59,66,67].

Regarding gut microbial metabolism, despite the fact that OO and OBs have been found to be mediated by the action of microbiota of the intestinal epithelium and suffer gut catabolism [68], the literature’s data on metabolism and bioavailability in the colon are scarce [69]. In vitro fermentation models have weaknesses, mainly their limited representativeness of in vivo conditions (e.g., colonocyte absorption, mucosa-associated microbiota and changes in physiological conditions during the transit time). Nevertheless, they are useful assays to explore gut microbiota catabolic activity on parent compounds and investigate their metabolites. In 2014, Mosele et al. [52] studied the colonic metabolism of HTyr with an in vitro model using human fecal microbiota. HTyr showed partial degradation until 6 h of anaerobic incubation with human gut bacteria and remained stable until 48 h of fermentation. Three metabolites, 2-(3′,4′- dihydroxyphenyl) acetic acid commonly known as DOPAC, 2-(4′-hydroxyphenyl) acetic acid and phenylacetic acid (PAA) were identified as major metabolic derivatives. These metabolites are involved in dopamine and tyrosine biosynthetic pathways and are generally considered metabolites produced in parallel to HTyr metabolism [70]. PAA was the main end catabolite through the in vitro fermentation with colon bacteria of HTyr, reaching a peak at 24 h [52]. Additionally, in a recent study, an in vitro gastrointestinal dialysis-colon (GIDM-colon) model—which is a continuous flow in vitro dialysis system simulating absorption from the lumen to the mucosa, followed by the colon phase—was used to explore the metabolism of HTyr as a pure compound [71]. Authors found that the catechol group of HTyr played a key role in its metabolic fate. They noted that the ortho–hydroxyl group of HTyr seems to promote autooxidation reactions through the formation of ortho–quinones, which trigger a sequential chain of reactions leading to a variety of metabolites. In total, 27 metabolic derivatives were tentatively identified, including esters, oxidized, methylated, dehydrogenated and dehydroxylated derivatives. Another important finding of this study is the detection of HTyr dimers and trimers and the proposal for the first time of auto-oxidation as the leading reaction of HTyr polymerization [71].

Overall, based on the existing in vitro data HTyr, is well absorbed across intestinal epithelial cell monolayers even though its lipophilic derivatives are significantly better absorbed and largely converted into free HTyr. This information could give rise to the development of analogues or natural pro-drugs with improved absorption characteristics. The existence of HTyr metabolic derivatives suggests the transformation activity from enterocytes and hepatic cells. HVAlc and its conjugates are considered the main metabolites detected using enterocytes. Being also endogenous metabolites, it seems that the presence of HTyr might participate in their regulation in human organism. Within this line, extensive uptake and metabolism of HTyr is observed using HepG2 cells as a model system of the human liver. In this case, glucuronide, methyl, sulfate and methylglucuronide conjugates are the main metabolites reported so far. Fecal fermentation models indicate that the metabolism of HTyr by gut microbiota occurs firstly by oxidation, followed by a transformation into hydroxylated phenylacetic acids, and finally autoxidation of the ortho-hydroxyl-group has been reported as the leading reaction which triggers the generation of HTyr metabolic derivatives as well as di- and trimers production.

#### 3.2.2. In Vitro Bioavailability and Metabolism Assays of Tyrosol (Tyr)

As mentioned already, Tyr differs in one hydroxyl group from HTyr, yet their bioavailabilities are quite dissimilar. In 2010, Soler et al. [58] investigated the metabolism of Tyr by intestinal epithelial cells and its transport across epithelial cell monolayers. Incubation of Caco-2/TC7 cells with Tyr resulted in slow conjugation reactions; the methyl and sulfate conjugates were only quantifiable after 24 h of incubation, showing similar metabolism yields to HTyr. Corona et al. [57] studied the fate of Tyr, along with other olive polyphenols, in the GI tract. Tyr was transferred across human Caco-2 cell monolayers and segments of rat jejunum and ileum and was subjected to classic phase I/II biotransformation. The major metabolites identified were glucuronides of Tyr—while no methylation occurred, in contrast to HTyr metabolites presented in the same study—probably caused by HTyr catechol moiety [57]. Regarding liver metabolism, when Tyr was tested in HepG2 cells, it was poorly metabolized, with less than 10% of the phenol glucuronidated after 18 h [66].

As for colonic metabolism, Tyr has been introduced as part of phenol-enriched OO to an in vitro colonic fermentation model, resulting in 2-(4′-hydroxyphenyl) acetic acid, which was the only fermentation product detected, and its concentration was relatively low after 48 h of incubation [52]. Tyr showed a partial degradation until 6 h and remain relative stable at 48 h of fermentation, but in lower concentration than its metabolite. As a general trend, Tyr showed a rise in the production of PAA and phenylpropionic acids and their hydroxylated forms in fecal culture mediums over up to 48 h of fermentation, reaching their maximum concentration between 12 and 48 h [52]. These results follow the same lines with HTyr in vitro metabolism, for which the same compounds have been detected in similar concentrations [70], though bioaccessibility was not a parameter explored in the case of Tyr. The in vitro GIDM-colon has been also employed to explore Tyr bioavailability, revealing that Tyr metabolites are degraded by the microflora of the colon in a similar manner as HTyr [71]. Overall, Tyr, compared to HTyr, seems less prone to biotransformation and although it shows good permeability performance, it has been found to suffer fewer metabolism reactions, possibly due to the lack of the extra hydroxyl group and the absence of the catechol moiety of the molecule.

#### 3.2.3. In Vivo Assays for ADMET Properties of Hydroxytyrosol (HTyr)

Several in vivo assays have been reported for the study of HTyr absorption mechanisms. In 2001, Visioli et al. [72] quantified HTyr in the urine of male rats via GC-MS after oral administration of three different doses of an OO mill waste water extract rich in HTyr (41.4, 207 and 414 μg/kg). Their results disclosed that HTyr is dose-dependently absorbed and excreted in urine primarily as a glucuronide conjugate [72]. Additional PK profile experiments have shown that after intravenous injection of pure radiolabeled HTyr (^14^C) in rats, it is quickly absorbed. Over 90% of the administered radioactivity is excreted in urine after 5 h, indicating that renal excretion represents the preferred path of excretion, while only 5% of radioactivity was observed in feces and the GI tract [70]. However, more recent experimental data suggest that oral administration of HTyr and its derivatives in higher doses (1 and 5 mg/kg) do not provide a linear, dose-dependent plasma concentration or urinary excretion in rats. Authors proposed that HTyr seems to reach a saturation point with respect to the dose above which no more absorption occurs, which can be critically affected by the saturation of phase I metabolic processes of intestinal transporters [73]. It is worth noting here the different HTyr kinetics observed based on the above studies depend on the administration route as well as the purity of the administrated compound, which possibly indicates the interference of co-existing compounds.

Fewer studies have been conducted focusing on HTyr plasma concentration due to detection difficulties in plasma. Studies have shown that HTyr has a swift absorption process and approaches C_max_ in plasma about 7 min after oral administration of 10 mg HTyr and plasma analysis via GC-MS [74]. In other studies, authors claim that the maximum plasma concentration of HTyr is reached between 0.5 and 1.0 h after its oral administration and that after 4 h, it is practically undetectable via UPLC-MS/MS [73]. According to recently published articles, gender has been uncovered as a critical feature for the bioavailability of HTyr. It has been observed that after oral administration of HTyr, it persists in the body of female rats for a longer period of time [73]. The study of the differential excretion by male and female rats under the same treatment revealed a critical influence of the sex-linked metabolism on the excretion pattern. Whilst the C_max_ in both males and females reach maximum in approximately the same period of time (between 0.5 and 1.0 h after administration), differences occur in urinary concentrations of HTyr and its metabolic derivatives in male and female rats. Results emphasize the influence of sex parameters on enterohepatic circulation, which is further confirmed by the presence of significant plasma concentrations 120 min after administration, indicating a longer presence in female organisms prior to excretion [73].

HTyr, after its absorption, seems to undergo a rapid and intense metabolism in animals. In brief, HTyr is initially subjected to phase I metabolism inside enterocytes and then recirculates in the liver. Afterwards, it enters the systemic and enterohepatic circulation and finally reaches the large intestine. In that level, gut microbiota are the responsible modulators for absorption and biotransformation of HTyr and its metabolites [12]. Several in vivo assays have been performed towards the exploration of HTyr metabolism and tissue distribution. Usually, UPLC-MS/MS methodologies are incorporated to facilitate metabolites identification in plasma, urine and tissues [75]. Regarding phase I metabolites of HTyr, oxidized derivatives have been reported, namely DOPAC and 3,4-dihydroxyphenylacetaldehyde (DOPAL), which are considered non-specific since they are also naturally produced through the dopamine pathway. During phase II reactions, *O*-methylated forms, specifically homovanillic acid (HVA) and HVAlc, have been detected. HTyr-1-acetate, a metabolite also found in OO, has also been found to be produced under alkaline lumen conditions. This metabolite can be further biotransformed into HTyr-1-acetate-4′-*O*-sulfate [76]. Moreover, N-acetyl-5-S-cysteinyl-HTyr has been identified in rats, derived from the autoxidation of HTyr to a quinone form. In the subsequent reaction with glutathione (GSH), it produces the conjugate, which is cleaved to the final metabolite by the enzymes *γ*-glutamyl transpeptidase (GGT) and N-acetyl transferase (NAT). The formation of the mercapturate conjugate of HTyr has been found to be dose-dependent [77].

The incorporation of animal studies for the exploration of a compound bioavailability and tissue distribution is a critical point since no other approaches (neither in vitro nor human) can offer the analysis of tissues. Beginning with the stomach, in most studies, oxidation and methylation reactions have been reported to occur, leading to the formation of phase I and II metabolic products (DOPAC, HVAlc and HVA) [75]. Additionally, some authors claim that the existence of SULT1C2 isoform in stomach tissue engenders sulfation reaction and imminent detection of the sulfated (sulf) forms of the already-metabolized compounds (HVA-sulf and HVAlc- sulf) [75]. However, other studies underline that sulfation can be carried out exclusively in the liver and that only phase I metabolism reactions occur in the stomach [78]. The detection of higher concentrations of phase I and II conjugated metabolites in the small and large intestine in comparison to the stomach indicates the higher level of enzymatic activity in the intestinal epithelium with an efflux transport of the metabolites into the gut lumen, a phenomenon that has been described for OO phenolic compounds [70,75]. HVA-sulf, as well as its glucuronide (glu)-conjugated form (HVA-glu), has been detected in the intestinal lumen in their highest concentrations [75]. In plasma, free HTyr has been detected in quite low concentrations and mainly in its metabolized forms: HVA, HVAlc and the respective glucuronide and sulfate conjugates [12,79,80].

**Figure 3:**
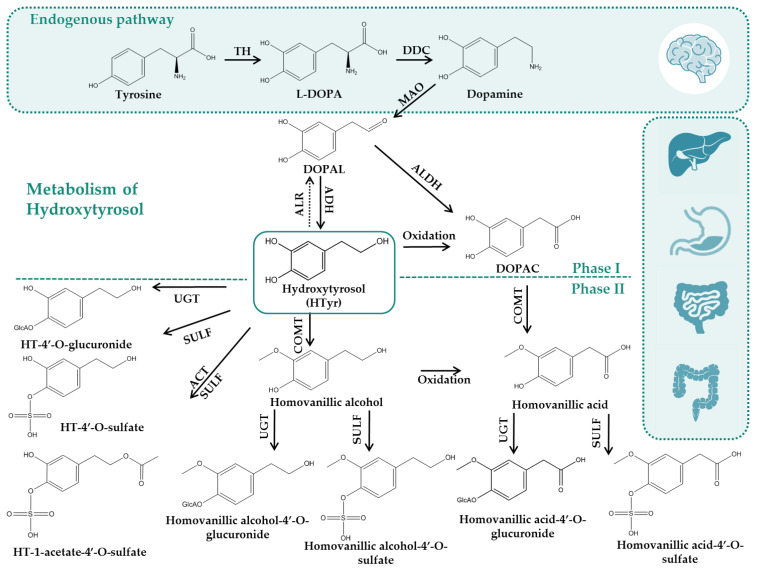
Biosynthesis and metabolism of hydroxytyrosol (HTyr) in in vivo systems. TH: tyrosine hydroxylase, DDC: dopa decarboxylase, MAO: monoaminoxidase, ALR: aldehyde reductase, ADH: alcohol dehydrogenase, ALDH: aldehyde dehydrogenase, COMT: catechol-O-methyl transferase, UGT: uridine 5′-diphosphoglucuronosyl transferases, SULF: sulfotransferase, ACT: O-acetyltransferase.

Studies investigating the distribution of phenolic compounds to rats have shown that HTyr and its metabolites have very good distribution abilities to tissues, including muscles, testis, liver, spleen, heart and brain, and are mainly accumulated in kidneys and the liver [81]. It is notable that renal uptake has been found to be 10 times higher than that of other organs. Additional studies after oral administration of increasing doses of HTyr (1, 10 and 100 mg/kg) to rats (given in a refined OO matrix) reassert that HTyr is accumulated in a dose-dependent manner not only in urine and plasma, but also in the liver, kidney and brain [82]. Kidneys and liver have been found as the organs possessing the highest uptake of HTyr and its metabolites. HTyr-acetate and HTyr-acetate-4′-*O*-sulf, due to their more lipophilic character in comparison to HTyr, have been found to more easily cross the bilayer membrane at the intestinal level and in this sense, the highest plasma concentration of HTyr has been found to persist longer between 0.5 and 2 h after oral administration when HTyr is administered in an acetate form [73]. As has been mentioned, it has been confirmed that HTyr, HTyr-sulf, HTyr-Acetate-sulf and Tyr (as dihydroxylation product of HTyr metabolism), are capable of crossing the blood–brain barrier in rats [75], though detected in low concentration (nM range), probably due to the low administered dietary doses in such experiments [76] Figure 3 presents schematically the metabolism of HTyr based on the available information so far. It is notable that HTyr is the only olive compound that has been found to be a dopaminergic neuronal protector not only due to its ability to cross the blood–brain barrier but additionally for its potential interactions with dopaminergic pathways [70,81] after studies in rodents associating HTyr consumption with brain protective effects [76]. However, it must be noted that detection of HTyr in the brain tissue could be also achieved naturally in low concentrations as a product of dopamine oxidative metabolism [83]. A common issue in brain in vivo models is proving the increase in HTyr and/or its metabolites in brain, taking into consideration the naturally found brain concentration and associate HTyr and its metabolites either after compounds’ ingestion or supplementation [76]. A targeted study focusing on the analysis of HTyr metabolites brain accumulation after a rat was supplemented with HTyr and its precursors (Oleu and Oleu aglycone) at a dose of 5 mg phenol/kg/day for 21 days confirmed that the main HTyr-circulating metabolites may have biologically significant activity within the brain [76]. Another interesting observation regarding HTyr metabolism is that the highest levels of urinary HTyr reported so far have been achieved after the ingestion of wine with ethanol. The reductive environment generated during alcohol metabolism is thought to be responsible for the change in DOPAL metabolism, enhancing the formation of the alcohol derivative, namely HTyr, instead of the acidic derivative DOPAC [12].

The dynamic introduction of HTyr as a promising component in food supplements and nutraceuticals raises the need for studies demonstrating HTyr toxicity. Toxicity is usually assessed in cells and animal models by determination of the acute toxicity, teratogenicity, mortality, morbidity or mutagenic effect [84,85,86]. Because of its low bioavailability and difficulties of its detection in plasma, few studies have been conducted to determine HTyr toxicity at high administered concentrations either as a pure compound or part of a nutritional supplement or enriched foods. Towards acute toxicity determination, D’Angelo et al. administered a single dose of 2 g/kg of body weight in rats and reported an absence of toxic effects or macroscopic alterations in organs. Only the appearance of piloerection was indicated at 2 h after administration, which disappeared in less than 48 h [70]. Nevertheless, subchronic toxicity is another issue in such studies [87]. For this purpose, in another study, authors used an extract called H35 (35% *w*/*w* HTyr), which was supplemented by oral gavage for 90 days at doses of 125, 250 and 500 mg HTyr/kg/day to female and male Wistar rats. No toxicologically significant treatment-related changes were observed in the condition or appearance of rats; neurobehavioral outcomes, motor activity assessments, functional observational battery, food intake, ophthalmoscopic examinations, hematology, clinical chemistry, urinalysis, necropsy findings and sperm parameters or estrus cycle were reported, and the only and lowest observed adverse effect level (LOAEL) was the 500 mg HTyr/kg bw/day based on statistically significant reductions in body weight gain and decreased body weight in males. According to the existing data and taking into account toxicity descriptions from studies carried out to other phenolic compounds and their high physicochemical similarities, scientists could approximate its null toxicity at all levels fairly accurately, although they underline that more studies to be done to substantiate this claim in humans [88].

#### 3.2.4. In Vivo Assays for ADMET Properties of Tyrosol (Tyr)

Regarding Tyr, less data is available for its absorption and metabolism. So far, there are no studies reporting the detection of Tyr when administered as a pure compound. Only one study has been carried out in rats reporting and monitoring a sulfoconjugate of Tyr and its distribution, verifying the compound’s absorption and low bioavailability [89]. Previous studies have shown that the majority of the OO phenolic compounds move unchanged through the mouth and stomach on their way to the small intestine and colon [90]. Unfortunately, the absorption of Tyr is poorly explored; no absorption rates have been established, and more studies are needed for the investigation of parameters affecting its absorption.

From the available studies so far, it seems that Tyr is subjected to an extensive metabolism in in vivo systems, and its bioavailability is low in respect to the metabolic derivatives attributed to Tyr metabolism [12]. At this point, it must be noted that studies investigating the metabolism of secoiridoids, which contain Tyr in their chemical structures (e.g., ligstroside, ligstroside aglycone), have shown that their hydrolysis leads to a three-fold increase in free Tyr after only 30 min in the acidic gastric environment [57]. This observation suggests that Tyr administration in secoiridoid form might be a more suitable precursor for an increase in Tyr bioavailability.

Within the few published data, a PK study of Tyr metabolites in rats gives insight to this phenylalcohol metabolism [89]. Blood samples were collected via the orbital venous plexus at various time intervals after a single orally administered dose (100 or 200 mg/kg) of pure Tyr as a suspension in polyethylene glycol and analyzed with UPLC-ESI/Q-TOF-MS. The authors reported two major metabolites and more specifically, they identified Tyr-4-sulf and a second metabolite that was not identified ([M-H]^-^ ion at *m*/*z* 151.0). However, other expected metabolic derivatives, such as glucuronides or methoxylated derivatives, were not detected, as in the case of HTyr. Regarding the time-dependent changes in the concentration of Tyr metabolites, the HPLC analysis of metabolites in plasma samples at different time points (15, 30, 60, 120 and 240 min) showed that they are rapidly absorbed, as evidenced by the mean plasma concentration–time profiles and their absorption gradually decreased at later time points. According to the authors, the results indicate that Tyr metabolites are formed shortly after oral administration in rats and might be absorbed directly through the stomach wall. Tissue analysis revealed that Tyr-4-sulf was the major metabolic derivative, which was detected 1 h after oral administration and accumulated in the kidneys and liver. The compound was not detected in epididymal fat or the lungs, indicating that the metabolite is mainly excreted by the kidneys through the liver [89]. It is very interesting that Tyr also exists in a glycoside form known as salidroside (*p*-hydroxyphenylethyl-*O*-*β*-D-glucopyranoside), found in OO by-products, which has been studied for its PK properties [91]. Salidroside was administered as a pure compound dissolved in saline through the vena caudalis at 50 mg/kg body weight. After salidroside hydrolysis and Tyr generation, authors found that Tyr underwent a rapid and wide distribution in rats’ tissues within 4 h of administration. The maximum concentration was found at 0.17 h. However, the maximum concentration could be higher and may be achieved earlier than the time point used in the study. Its maximum levels were detected in the heart, followed by the spleen, kidney, liver and lungs, in that order. Additionally, Tyr showed a substantial distribution in the brain and demonstrated its ability to cross the blood–brain barrier, similarly to HTyr. After 0.17 h of administration, the concentrations of Tyr declined abruptly and was almost undetectable after 4 h of administration [91]. The same elimination time has also been estimated for HTyr [73].

From all of the above, it can be assumed that phenylalcohols are compounds that are well absorbed in biological systems and have high distribution potency. Based on studies investigating OO phenolics, it seems that Tyr and HTyr have been demonstrated to be the best-absorbed compounds in the intestinal tract (absorption rate ≈ 40–95%) in a dose-dependent manner [4,27]. Both compounds have been detected in most animals’ tissues, including the brain, they are accumulated in the liver and kidneys and are undetectable 4 h after administration. HTyr is more extensively studied concerning its metabolism in comparison to Tyr and more metabolic derivatives have been identified. For both compounds, sulfation has been revealed as the major metabolic reaction and the respective metabolic derivatives are accumulated in the liver. Although HTyr and Tyr data are generally in agreement, more studies are needed for the exploration of Tyr metabolism.

### 3.3. ADMET of Secoiridoids Oleacein (Olea), Oleocanthal (Oleo) and Oleuropein (Oleu)

Olea, Oleo and Oleu are olive secoiridoids, belonging to OBs, with significant pharmacological properties and interesting physicochemical characteristics. Based on the literature, it can be hypothesized that a positive association exists between these compounds and the beneficial effects of olive products, whose consumption is directly associated to MD, advanced life expectancy and reduced incidences of age-related diseases [92].

Olea is a dialdehydic derivative of decarboxymethyl elenolic acid (EDA) bound to HTyr (3,4 -DHPEA-EDA). Olea is very similar to Oleo since the latter differentiates in one less -OH group at C-3 of the phenylalcohol part. Olea and Oleo, the OO secoiridoids, have drawn scientific attention in recent years, mostly after a publication denoting Oleo’s anti-inflammatory activity and comparing it to ibuprofen, a commonly used analgesic and anti-inflammatory agent [93]. A considerable amount of research has been devoted since then to the investigation of their pharmacological properties, leading to the discovery of significant properties and promising results [53,94,95]. However, their metabolism, bioavailability and PK characteristics in in vivo models and human systems are poorly studied.

This fact could be considered relatively unexpected since Olea and Oleo, with Oleu aglycones and Lig aglycones, comprise the major secoiridoids of OO. One reason could be the chemical nature of these compounds being an ester associating the HTyr/Tyr part with EDA (Figure 1). Due to their structure, they are expected to be rapidly hydrolyzed, giving rise to HTyr and Tyr and their metabolites [75,89,96]. One other reason could be connected to an extra chemical feature of Olea and Oleo. Their molecular scaffold is characterized by highly reactive hydrogens belonging to the two aldehydes rendering the molecules prone to equilibria and therefore sensitive and unstable. Consequently, their isolation and purification is a laborious procedure, usually with multiple chromatographic steps resulting finally in the unavailability of reference standards until recently [57]. Additionally, their labile nature makes their detection in biological fluids and tissues a challenging analytical task [46]. Overall, these facts impede the performance of in vivo and clinical experiments due to the high amounts of pure compounds required for such studies, rendering scarce the metabolism data of these compounds in biological systems.

Oleu is characterized as a nontoxic natural secoiridoid comprising the main phenolic compound in *Olea europea* L. (Oleaceae) and is found in almost all olive products and by-products, with the exception of OO, in which it is detected in traces [97]. Oleu is a glucoside ester of EA and HTyr with diverse, extensively studied health benefits, such as antioxidant, cholesterol lowering, cardioprotective, anti-inflammatory, hypoglycemic and antimicrobial properties [24,98,99,100,101]. Despite Oleu being determined in plasma [102,103], this compound undergoes extensive metabolism to HTyr and other products, such as EA, HTyr, Tyr and glucose [104].

The structural similarities of Olea, Oleo and Oleu are likely responsible for the remarkable pharmacological properties thereof. Despite the plethora of literature investigating their significance for human health, the structural peculiarities of these secoiridoids are partially responsible for the gap of reports investigating their bioavailability in biological systems and more research is needed to fill this gap. Tables S1 and S2 summarize the existing bibliographical data regarding their bioavailability and metabolism investigation through in vitro assays and in vivo animal studies discussed in detail in the next paragraphs.

#### 3.3.1. In Vitro Bioavailability and Metabolism Assays of Oleacein (Olea), Oleocanthal (Oleo) and Oleuropein (Oleu)

Sporadic information exists in the literature for Olea, Oleo and Oleu in vitro bioavailability and assays mainly concern physicochemical observations being useful in ADME(T) investigation. It has been reported that Olea may be absorbed in the small intestine by passive diffusion through the membrane due to its favorable partition coefficient (log *p*  =  1.02). Contrary data exist in the literature for the stability of Olea in gastric conditions. In a recent study by Reboredo-Rodríguez et. al., Olea was found to be extensively hydrolyzed [105], while in a previous study, Olea was found to be stable at gastric acid pH and 67% remaining unchanged after 4 h of incubation [90]. Oleu, on the other hand, has been investigated for its intestinal absorption via in situ intestinal perfusion with isolated rat intestine, which resulted in poor absorption with the mechanism not being able to be defined [106]. Another study supported that glucose transporter in the epithelial cells of the small intestine is most probably involved in Oleu’s absorption mechanism [107]. Unlike Oleu, its corresponding aglycone (3,4-DHPEA-EA, Oleu aglycone) may be more effectively absorbed in the small intestine due to its higher partition coefficient (log p) value [108,109].

The most prevailing theory for the metabolic fate of Oleu is that it is stable in the stomach, it is not absorbed or metabolized in the small intestine, and it is likely to reach the large intestine, where biotransformation occurs by gut microbiota to finally yield HTyr, which may then be absorbed in the large intestine [104]. Specifically, it is well established that in vitro deglycosylation of Oleu, yielding the aglycone forms, is readily achieved by *β*-glycosidases in various pH treatments. Sequentially, cleavage of aglycone’s ester bond produces HTyr. Mosele et al. [52], during in vitro colon fermentation of Oleu, reported high degradation of the parent compound, an increase in phenolic acids and the stability of HTyr as a deconjugation product of Oleu. However, it is important to note that the metabolic fate of the elenolic part of Oleu is totally neglected so far.

Additionally, fewer data exist for Oleu aglycone. Oleu aglycone has been found to be relatively stable under acidic in vitro gastric conditions, suffering limited hydrolysis, leading to the increase of HTyr only 30 min after incubation in an in vitro rat intestinal model [110]. The compound seems to pass across a human cellular model of the intestine (Caco-2 cells), while no glucuronide conjugation was observed although HTyr and HVAlc were formed in low yields. Additionally, it was reported that Oleu aglycone undergoes extensive metabolism under reduction and glucuronidation during the pass across both the ileum and jejunum in isolated perfused segments of intestine. In agreement with the cell studies, perfusion of the jejunum and ileum also yielded HTyr and HVAlc and their respective glucuronides. The authors support that the reduced and glucuronidated forms represent novel physiological metabolites of the secoiridoids that should be pursued in vivo [110]. The impact of both gastric and small-intestinal phases of digestion on OO phenols was examined, and the major OO phenolics (Tyr, HTyr) and the related secoiridoid (Olea and Oleo) showed good stability in the gastric digestion model [58]. In contrast, the stability of these dietary phenols when exposed to small-intestinal conditions (incubation at pH 6.5 with pancreatin and bile salts at 37 °C for 2 h) was very low. In fact, from the main phenols of virgin OO, only 10% of the secoiridoids Olea and Oleo were recovered [58].

#### 3.3.2. In Vivo Assays of Oleacein (Olea), Oleocanthal (Oleo) and Oleuropein (Oleu)

In general, in vivo studies have shown that OO phenols are dose-dependently absorbed and excreted in urine either in their free form or as conjugated metabolites [111]. Contrary to phenylalcohols, for olive secoiridoids there is a serious lack of in vivo reports arising from the respective lack of methodologies in the literature for their determination in biological matrices [106]. So far, the data for the in vivo absorption and bioavailability for olive secoiridoids are scarce.

Regarding Olea, in a study where it was administered orally and intravenously in pure form in doses of 300 mg/kg and 10 mg/kg, respectively, it was not detected at all in plasma in its parent form [112], an observation in accordance with the in vitro assays. Authors denoted that the existence of the two aldehydes in the molecule might cause the quick binding of the molecule to proteins bearing primary amines, such as globulins, albumins and other functional proteins, in blood plasma [112], a phenomenon that was previously described for HTyr as well. In the same study, Oleu was administered orally and intravenously in the same manner as Olea. The compound was detected 5 min after its oral administration. However, it was detected only in low quantities and consequently was not quantified [112]. HTyr, HVAlc, and HVA were detected as metabolic derivatives for both compounds, indicating the absorption as well as the high lability and rapid degradation of this class of compounds. The absorption rate of Olea was found to be 13.5 times greater than that of Oleu [112]. To the contrary, in another study, Oleu was detected in plasma 10 min after oral administration and the maximum peak value was reached at 2 h [102]. In urine, Oleu and Oleu aglycone can be detected within 4 h in their free forms. However, they do not exceed 15% of the initial administered dose [113]. As can be assumed, more data are needed for the determination of Oleu in plasma and the development of a sensitive and selective methodology for the detection of olive secoiridoids in plasma samples.

**Figure 4:**
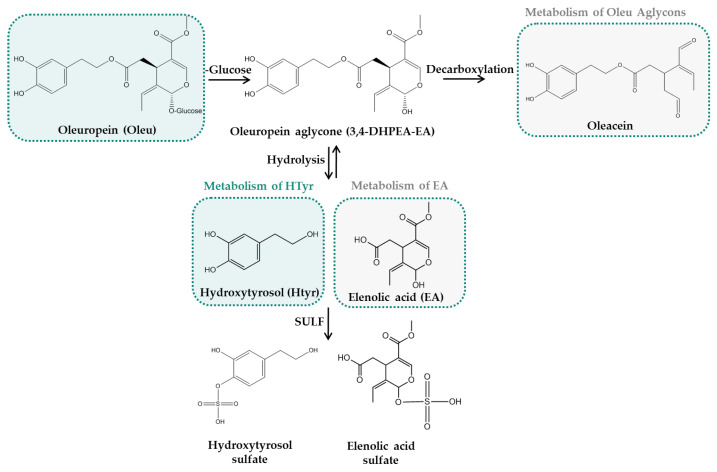
Metabolism of oleuropein (Oleu) in in vivo systems. The gray squares represent the parts of secoiridoids that are neglected so far.

Likewise, more data are available regarding Oleu metabolism, although its supplementation mainly occurs via enriched extracts. It has been found that Oleu undergoes extensive non-enzymatic hydrolysis by gastric juice or decomposition by colon microflora, forming HTyr, which enters the small intestine and is subsequently absorbed by passive diffusion or by the colon [113]. HTyr and EA glucoside are the first derivatives of Oleu derived from the hydrolysis of the ester bond [102], with HTyr being recognized as the major derivative of Oleu and Oleu aglycones as well [112]. HVA and HVAlc have also been identified as Oleu and Oleu aglycone metabolic derivatives. These metabolites comprise the major phase I metabolic derivatives of Oleu, which could be further metabolized to phase II derivatives, generating the respective sulfate, glucuronide and other conjugated forms [112]. Unfortunately, as previously mentioned, Oleu is usually administered in the form of enriched extracts, and no data were found for the phase II metabolism and tissue distribution of pure administered Oleu in in vivo systems. More studies are required to come to conclusions about its metabolic derivatives. Furthermore, the bioavailability of OO phenolic compounds depends on the carrier by which they are administered (oily or aqueous) as well on the administration route (intravenous or oral) [114]. Figure 4 briefly presents the metabolism of Oleu.

In the few existing studies, Lopez de las Hazas et al. [75] investigated and compared the absorption, metabolism and subsequently the tissue distribution and excretion of HTyr administered either in its free form or through its esterified precursors, Oleu and Oleu aglycone, in rats. Subjects were fed a diet supplemented with the equivalent of 5 mg phenol/kg/day for 21 days, and the HTyr metabolites in the GI tract (stomach, small intestine and cecum), plasma, urine and metabolic tissues (liver and kidney) were analyzed. Compared to HTyr and Oleu aglycone, Oleu showed greater stability during digestion, resisting in part the action of the *β*-glucosidase enzymes in the small intestine and passed unaltered to the colon with the resultant higher production of microbial fermentation catabolites. Consequently, the bioavailability based on the urine excretion of HTyr metabolites was higher. Accordingly, previous studies have shown that Oleu is stable in human gastric juice, while Oleu aglycone has been shown to be more sensitive to temperature, pH and enzyme activity [75]. According to this study Oleu, could suffer less degradation than HTyr and Oleu aglycone under alkaline intestinal conditions and therefore, it could have been more exposed to phase II metabolism. In another study, rat feces and urine were analyzed for the investigation of the in vivo bio-transformation of Oleu after its oral administration (100 mg/kg) dissolved in 1 mL water [115]. Samples were collected from 0 to 24 h and then analyzed by LC-MS/MS. According to the metabolites analyzed in rat feces, the authors proposed a metabolic pathway of Oleu in the GI tract. Specifically, Oleu could be hydrolyzed by acidic condition in the stomach or by *β*-glycosidase in the intestinal tract to produce Oleu aglycone and glucose. Subsequently, the aglycone is hydrolyzed to EA and HTyr and then HTyr is transformed into HVA. Analysis of metabolites in urine samples indicated that the parent molecule Oleu and its three metabolites (Oleu glycone, EA and HTyr) were in systemic circulation within 24 h after oral administration to rats. This was the first report showing that Oleu aglycone can be found in rat feces and urine after oral administration, indicating that Oleu aglycone may play an important role in Oleu bioactivity. Contrary to this observation, in vitro results have shown that Oleu aglycone is unstable and easily biotransformed to HTyr. In a similar study, the same group investigated the bio-transformation of Oleu in rats after intravenous administration and found one mono-oxygenated metabolite [116]. Oxygenation was found to be the major metabolic process of Oleu in rat blood circulatory system after intravenous administration [116].

In spite of the strong evidence for the positive impact of Oleo and Olea on human health [117], as was previously mentioned, the unavailability of commercially available standard compounds confines the investigation in in vivo systems and the study of their absorption and metabolism. Few experimental protocols have been proposed for their detection in biological systems, and until now, these two compounds have not yet been detected, or contradictory results are generated due to the absence of an appropriate and sensitive methodology for their detection [46,118,119].

Two recently published studies exploring the in vivo intestinal absorption, metabolic profile and tissue distribution of Oleo in rats were found [118,119]. At the first approach, an in situ perfusion technique in rats was used, involving simultaneous sampling from the luminal perfusate and mesenteric blood. Samples were analyzed using UHPLC–MS/MS for the presence of Oleo and its metabolites. The authors detected Oleo in plasma and lumen and identified four metabolites in plasma (Oleo + OH, Oleo + H_2_O, Oleo + H_2_ + glucuronide, Oleo + H_2_O + glucuronide) and two metabolites (hydroxylated and hydrated) in perfusion samples. The authors estimated that the relative abundance of metabolites was higher in plasma than in the lumen by calculating the ratio of metabolites/Oleo but without commenting on the method of Oleo peak area calculation. Between the two metabolites corresponding to phase I metabolism (hydration and hydroxylation), the most abundant was the hydrated form, with higher levels in plasma than in the lumen (*p* < 0.05) [118]. Authors referred that the metabolites in the lumen can originate from the microbiome and metabolic reactions caused from enzymes present in the epithelium of the small intestine [120] and subsequently, the absorbed and metabolized Oleo would be secreted to the intestinal lumen by efflux transporters. The authors also commented that the redox potential in the intestine favors the reduction reaction due to low oxygen tension, which provides a reducing environment, whereas oxidation is favored in tissues such as the liver. The authors concluded that Oleo is a phenolic compound with low oral bioavailability (16%) due to its high intestinal metabolism. Its poor absorption was indicated by a low effective permeability coefficient, apparent permeability coefficient and area under the mesenteric blood–time curve normalized by the inlet concentration in comparison with the reference standard compound [118].

In the second and most recent study, the distribution of Oleo and its metabolites was examined after the acute intake of refined OO containing 0.3 mg/mL Oleo [119]. Plasma samples, along with heart, liver, spleen, lung, kidney, brain, thyroid, stomach, small intestine and skin samples, were collected at three different time points—1, 2 and 4.5 h after administration—and then analyzed via UPLC-ESI-LTQ-Orbitrap MS. Ten phase I and II metabolites were identified (Tyr, Oleo + H_2_, Oleo + OH, Oleo + OH + H_2_O, Oleo + H_2_O, Oleo + OH + CH_3_, Oleo + H_2_O + CH_3_, Oleo + OH + glu, Oleo + H_2_O + glu, Oleo + OH + CH_3_ + glu), many of them in common with the previous study. Contrary to the previous study, Oleo was not detected in plasma, though in this case, the compound was administered by oral gavage and not directly to the small intestine, avoiding passing through the stomach and liver [118]. However, the authors supported that a possible explanation for Oleo absence from plasma in their approach could be the relatively low absorption (16%) and high intestinal metabolism of Oleo [118]. Oleo was detected only in the stomach and the small intestine, presenting maximum concentration at 1 h and decreasing in concentration with time. Based on authors results, Oleo is hydrolyzed in the stomach to Tyr and the non-hydrolyzable Oleo undergo further metabolic reactions. Oleo metabolites were widely distributed in all analyzed tissues, even in brain and skin, while the liver, small intestine and plasma were the biological samples with the greatest variety of metabolites. Oleo + OH + CH_3_ was found as the main circulating metabolite, followed by Oleo + H_2_O + glu. Kidney was the tissue with the widest range of metabolites, indicating that renal excretion is the major pathway of elimination of Oleo metabolites in rats, whereas skin revealed the fewest metabolites [119].

Regarding Olea, a recent in vivo study investigated its intestinal permeation and metabolism in rats. Specifically, mice were supplemented by a single-pass intestinal perfusion (SPIP) system at a dose of 60 mg/kg pure Olea. Plasma samples from mesenteric blood and samples from intestinal lumen were obtained. Olea was detected in plasma samples along with four phase I metabolites and six phase II metabolites. According to this study, metabolite levels were much higher in plasma than the lumen, similarly Oleo in the study by López-yerena et al. [118]. The main phase I metabolite both in plasma and lumen were HTyr and Olea + OH, reaching their maximum concentration at 55 and 60 min respectively. It has to be noted that EA was not detected as an Olea metabolic derivative despite the fact that hydrolysis has been reported as the main reaction of phase I metabolism. Based on the authors’ observations, the absence of EA could be explained by its rapid absorption. However, similarly to the case of Oleu, which has a very close chemical structure to Olea, HTyr represented the major metabolic derivative in plasma [121].

### 3.4. Human Trials for Phenylalcohols and Secoiridoids

Regarding human studies, there is a great number of reports investigating the metabolism of OBs. In most studies, OBs are supplemented in the form of enriched extracts, enriched OO or other enriched matrices. OBs are scarcely studied as pure compounds, and usually, ease of extract preparation along with isolation difficulties, especially in the case of secoiridoids, promote their study in the form of mixtures. HTyr and Oleu are prominent in most such studies and they are the only olive constituents that have been supplemented as pure compounds due to their higher availability as commercial standard compounds in comparison to Oleo and Olea as well as their safety, which to some extents has been substantiated. Tyr has generally fewer studies due to its lower bioactivity according to the available information. Table 2 below summarizes the human studies included in the current review, discussing the metabolism of olive bioactive constituents.

Starting with the most studied olive compound, the metabolism of HTyr was firstly elucidated in humans by Visioli et al. [111]. In this study, HTyr was administered in different matrices (OO and yogurt) and the excretion of HTyr and its major metabolites was determined in rats and humans, highlighting the differences in HTyr metabolism and urinary excretion in animal species. The results revealed that the urinary excretion of HTyr was greater in humans than in rats, indicating their different absorption rates possible due to the absence of a gallbladder in these rodents and pinpointing that rats are not an appropriate model for the study of HTyr metabolism [111]. Several studies have been performed since then for the determination of HTyr absorption in humans after its oral intake. Detection difficulties in plasma and the different protocols found in bibliography for HTyr plasma recovery have hindered the establishment of absorption rates for this compound despite the long research for the exploration of its metabolism. In most studies absorption rate ranges from 55–90% and absorption is usually confirmed by the excretion of HTyr and its metabolic derivatives in urine [122,130]. HTyr is present in plasma and urine mostly in conjugated forms as glucuronide and sulfate derivatives, suggesting an extensive first-pass intestinal/hepatic metabolism. Independently of the absorption rate, the time to reach plasma C_max_ has been determined to range in minutes from 10–30, depending on each individual and other factors, such as the supplementation matrix, which are discussed in detail in the next paragraphs [122,130]. Additionally, in all studies, it was reported that 1 h after HTyr administration, the molecule is completely undetectable [122]. Finally, after postprandial administration, HTyr binds to circulating human lipoproteins [139]. The plasma protein binding of HTyr is considered a critical topic of research not only from a PK perspective, but also due to its implications in drug–drug interactions; however, the plasma protein binding of OBs has not yet been adequately studied and more research is needed to obtain results and draw conclusions.

Metabolic derivatives of HTyr have not yet been fully described; new studies are continuously published and the number of the identified metabolites increases significantly year by year. More than 16 metabolites have been identified so far and some of them have been also characterized as HTyr biomarkers in human biological fluids (plasma or urine) when HTyr is administered as a food supplement or via OO consumption. Many of them are common in the in vivo observations, i.e., HVA, HVAlc, DOPAL, DOPAC, 3,4-dihidroxyphenylpropionic acid, protocatechuic acid. All of them produced—via oxidation and/or methylation of the aliphatic alcohol—conjugated metabolites, i.e., sulfates (HTyr-4′-*O*-sulf and HTyr-3′-*O*-sulf), glucuronides (HTyr-4′-*O*-glu and HTyr-3′-*O*-glu), acetates (HTyr-acetate) as well as all the above phase I metabolites in their sulfated and glucuronated forms (HTyr-1-acetate-4′-*O*-sulf, HVA-sulf, DOPAC-sulf, HVAlc-4′-*O*-glu etc) [46] have also been reported. The mercapturate conjugate of HTyr found in rats has not yet been identified in human biological fluids [77]. Evidently, tissue distribution cannot be determined. However, the substitution of the aromatic ring and the chemical groups of detected metabolites in biological fluids could witness the type of metabolic phase and their final percentage in urine could imply the most intense metabolism reaction. HTyr-sulf has been proposed as the most suitable biomarker for monitoring compliance in urine and plasma as well [132]. Nevertheless, other HTyr metabolic derivatives, mainly HVA and HTyr-glu, have been previously suggested and are still used as biomarkers [123]. In another recent study, 29 women with overweight/obesity received a soft capsule of 5 mg HTyr in two different doses (5 and 15 mg/day), and their urine metabolome was investigated to search for endogenous metabolites affected by long-term HTyr administration. Authors denoted for the first time the association of HTyr with endogenous compounds, such as hippuric acid and *p*-cresol derivatives [25]. Currently, many researchers are looking for new HTyr metabolic derivatives to enhance the knowledge about HTyr ADME(T) processes as well as their stability and health-beneficial properties [88].

Regarding elimination from the body, in humans, about 6 h are required for the complete elimination of HTyr and its metabolic derivatives, while in rats, as previously mentioned, the respective duration has been found to be approximately 4 h [12,124]. The biliary route transfers HTyr metabolites from the liver back into the duodenum, where they can be converted and reabsorbed. As a result, this enterohepatic recycling may lead to a longer presence of HTyr and metabolites within the body. HTyr is mainly accumulated in the kidneys until its excretion with urine [12,124]. HTyr, HVAlc, HVA and DOPAC have been identified in their free forms (44%) or as glucuronide (34.4%) or sulfate (21.2%) conjugates in the 24 h urine samples of subjects [42]. Urinary recovery of absorbed HTyr varied significantly between 5% and 72% of the content ingested, with approximately 90% excreted as conjugates in 24 h urine when OBs were administered as a supplement [51,134]. A study in which investigators reported the absence of HTyr in human urine after ingestion of an olive leaf extract by healthy young adults over 28 days or in a single bolus dose must also be quoted [129]. Additionally, they reported the existence of five Oleu-glu conjugated at different positions on the phenolic moiety [129]. However, HTyr absence from urine has not been reported by other authors. Similarly, conflicting data exist regarding the basic conjugates found in urine; some authors propose glucuronides as the major metabolites, while others propose sulfate derivatives [132,134].

Another interesting aspect in HTyr metabolism is that its endogenous metabolism is influenced by ethanol intake, a finding shown in animal studies as well [12]. In a double-blind, randomized, crossover, placebo-controlled clinical trial, the effect of ethanol dose on HTyr formation in humans was determined [133]. Twenty-four healthy male volunteers were split into three different cohorts and each one received two doses of ethanol or placebo. In total, six different doses of ethanol (6, 12, 18, 24, 30 and 42 g) were tested and urinary excretion of HTyr was assessed (from 0 to 6 h after administration). The amount of excreted HTyr increased as the ethanol dose was increased. HTyr-sulf was identified as the most intense metabolite of HTyr and in parallel HVAlc rose with the ethanol administered dose. Although this relationship was not found with HTyr-glu. Furthermore, in the same study, a reduction in the ratio DOPAC/HTyr from placebo to the highest dose (42 g of ethanol) was observed (from 14 to 3.6, respectively), consistent with the appearance of the shift in dopamine oxidative metabolism to preferentially produce HTyr rather than DOPAC [133].

Similarly to in vivo animal studies experiments, HTyr bioavailability is significantly influenced by several factors, such as age, hormonal status [131] or sex [127]. Among these factors, sex is regarded as a parameter of controversy and further studies are required to come to a conclusion. Women’s research was neglected until the last decade of the 20th century and results obtained in men were directly translated to women both in medicine and nutrition. Conflicting results exist in the literature for HTyr bioavailability and sex differences. In a study, authors conclude that there were no substantial variations in the levels of bioavailable HTyr after the oral intake of foods fortified with HTyr in relation to volunteers’ sex despite a higher, though non-significant, absorption and bioavailability in men compared to women [122]. J. Rodriguez-Morato et al. reviewed a subsample of PREDIMED study in which total HTyr concentrations were detected in urine after a traditional MD [140]. Ethanol consumption was taken also into consideration by analyzing ethyl glucuronide as a biomarker of alcohol intake. Covariance analysis (ANOVA) was conducted in total HTyr urine concentration on 341 males and 379 females adjusted for ethyl glucuronide as a continuous variable and sex as a categorical variable. The sex-related differences were found to be significant (*p* < 0.001), thus confirming the sex–gender dimorphism [141]. Another study, in which volunteers were supplemented with an olive leaf extract enriched in HTyr and Oleu, noted a large interindividual variation in absorption and metabolism of phenolic compounds, possibly resulting from differences in human enzymatic activity [127]. Males may be more efficient at conjugating Oleu, which would explain their lower AUC for Oleu but higher AUC for HTyr metabolites. Additionally, this study showed that conjugated metabolites of HTyr were the primary metabolites recovered in plasma and urine after Oleu ingestion. Peak Oleu concentrations in plasma were greater following ingestion of liquid than capsule preparations, but no such effect was observed for peak concentrations of conjugated HTyr (sulfated and glucuronidated).

Another parameter recently explored in humans is hormonal status [131]. A PK study exploring the presence of polyphenols extracted from olive leaves in plasma and urine was conducted and the metabolites in pre- and post-menopausal women were identified [131]. In this context, HPLC analysis indicated that most of the metabolites were conjugated, primarily as glucuronides and sulfates, while plasma and urine profiles were similar in both groups of women. The authors noticed the presence of 10 phenolic metabolites in common of among 15 identified compounds in the olive leaf extract. After 35–37 min, all these metabolites reached their maximum concentrations. These concentrations were substantially higher in post-menopausal women (i.e., HTyr-sulf presented the maximum concentrations in post-menopausal ≈ 128 μM) compared to pre-menopausal women (≈94 μM). Additonally, it was noted that the excretion of sulfated metabolites was not complete at 24 h after the intake of the olive leaf extract and that this fact could be related to their enterohepatic circulation. The presence of HTyr sulfoglucuronide in human plasma was also identified for the first time [131].

An additional parameter not yet explored in in vivo studies that is very important to consider in nutraceutical development is the vehicle of compound administration or used food matrix. Studies indicate that bioavailability is directly associated to a compound’s bioaccessibility framed through the matrix of administration and the absorption process depends on the used matrix for HTyr administration [122]. In this context, it was discovered that administration of HTyr in extra virgin OO resulted in better absorption in comparison to other oil matrices. Other matrices, such as water, yogurt or even adding it to refined OO, have shown significantly lower absorption rates [111]. The comparative analysis of urinary HTyr after the oral intake of the diverse fortified and non-fortified matrices (extra virgin OO, refined oil, pineapple juice, flax oil, margarine, seed oil grape) evidenced that the absorption and metabolic profile is affected significantly by the food matrix in which HTyr is incorporated, with the oily nature of the food matrix being more relevant for the bioavailability of this phenolic compound and extra virgin OO has been pinpointed as the best dietary source of this compound.

Since OO phenols are absorbed primarily in the small intestine and colon [134], the rate of gastric emptying and the slower release of HTyr from the oil matrix, which results in prolonged absorption rates [130], may be contributing factors [96,130], as compared to an aqueous matrix or a low-fat yogurt, a lipid matrix increases HTyr bioavailability [111]. Furthermore, the broad disparities observed in the absorption of HTyr when administered in virgin OO (with other polyphenols) compared to its pure form as an aqueous solution could be explained by the number of OBs, such as Oleu aglycone, that can release HTyr in the gut via hydrolysis [111]. The possible protection from binding to proteins in the gut or the presence of other OO antioxidants, which prevents the breakdown of phenols, may also result in increased bioavailability of HTyr in oil compared to aqueous solutions [111]. Additionally, it has been found that the bioaccessibility of HTyr is higher when phenol-enriched OO is administered with an OO enriched with its own phenolics plus additional phenolics from thyme [136]. Antioxidant spices, such as thyme, are thought to improve the bioaccessibility of HTyr derivatives by reducing their loss during the digestion process, which is consistent with previous findings [136]. De Bock et al. [127] estimated the bioavailability of Oleu and HTyr by measuring the phenolic content in the plasma and urine samples of healthy volunteers. The authors investigated the plasma and urine of nine subjects who received different doses of Oleu and HTyr in the form of capsule or liquid, observing higher concentrations of Oleu in plasma in the subjects who received liquid doses (2.74 ng/mL) compared to those who consumed capsules (0.47 ng/mL). In the same study, the authors also noticed that the primary metabolites identified in urine and plasma were the conjugated metabolites of HTyr [127].

In the literature, there are controversial results on the localization of OBs in LDL [48,125,126]. However, detection difficulties impede identification and subsequent compound quantitation. It is interesting that more than half of the HTyr detected in plasma is identified in the LDL-purified fractions when HTyr is administered as an aqueous supplement [42]. An interesting study by C. Fernández-Ávila et al. [137] assessed the influence of OO consumption on the phenolic metabolite levels in a European population based on a clinical intervention on 51 healthy men. In the study, an ultra-high-performance liquid chromatography (UHPLC) tandem mass spectrometry (MS/MS) method was used for the determination of specific phenolic compounds in human high-density lipoprotein (HDL) samples. The concentration of the studied metabolites (HTyr-sulf, HVA-sulf, HVA-glu) increased significantly after high-polyphenolic-content virgin OO ingestion (*p* < 0.05) compared to low-polyphenolic-content OO. It is interesting that among the metabolites that were studied, HTyr–sulf was the most abundant. The highest mean value for HTyr-sulf in HDL was 49.48 ng/mg APO-A1. Other similar studies with LDL samples found lower HTyr-sulf concentrations—about 24.27 ng/mg APO-B [135] and 34.22 ng/mg APO-B [48] 60 min after extra virgin OO intake. According to those authors, the most abundant metabolite in LDL was HVA-sulf [48,128,135]. In the above study, the mean value of HVA-sulf in HDL was 9.33 ng/mg APO-A1, while their findings showed a concentration of 17.0 ng/mg APO-B after the participants consumed virgin OO for three weeks. However, it should be mentioned that the obtained levels are being compared between different matrices, LDL and HDL lipoproteins, where the presence of metabolites is not necessarily equivalent. Generally, HTyr-sulf has been proposed and used as a biomarker for OO consumption in HDL [137].

Regarding the other secoiridoids, there are no reports for intervention studies in humans evaluating individual compounds or enriched extracts in these compounds. Studies for secoiridoids are mainly performed via supplementation of extra virgin OO or enriched phenolic extract of OO and data in the literature are limited. A recent interventional study on humans was published focused on the bioavailability and the interindividual variability of secoiridoids and their metabolites after an acute extra virgin OO intake [96]. Other typical phenolic compounds in extra virgin OO, such as phenolic acids, phenolic alcohols, flavonoids, lignans and their metabolites, were also monitored to ascertain whether any of these could also be used to study extra virgin OO intake. Nine healthy volunteers ingested 50 mL of extra virgin OO in a single dose containing 322 mg/kg total phenolic content (caffeic acid equivalents) and 6 mg of HTyr and its derivatives/20 g of extra virgin OO. Plasma and urine samples were collected prior and after ingestion (plasma: (0 h) and at 0.5, 1, 2, 4 and 6 h after ingestion and urine: (0 h) and at 0–4, 4–8, 8–15, and 15–24 h) and were analyzed by UPLC-HRMS. The authors applied partial least squares discriminant analysis with orthogonal signal correction (OSC-PLS-DA) to screen for metabolites that allow sample discrimination. Using this approach, three plasma secoiridoid compounds (EA + H_2_ detected at two retention times; Lig aglycone + H_2_ + glu) and nine urinary secoiridoid compounds (Olea, Olea + H_2_ + glu detected at four retention times, and methyl Olea + H_2_ + glu detected at four retention times) were selected as biomarkers for monitoring extra virgin OO intake in human intervention trials as they were discriminant for all time collection points when compared to baseline. It is interesting that similarly to the cases of HTyr and Oleu, the maximum concentration levels of the plasma biomarkers were detected between 0.5 and 2 h, and maximum excretion of urinary biomarkers occurred in the first 4 h after extra virgin OO intake. Although there were differences between rat and human microsomal activity, hydrogenation seems to be an important phase I reaction of metabolism for these secoiridoids. This evidence was supported by results obtained in the human intervention as hydrogenated metabolites were detected in plasma for EA. These results are in accordance with other similar studies investigating secoiridoid availability in human systems [124,138]. HTyr-sulf has also been highlighted as major biomarker of extra virgin OO intake, though authors underlined that absorption and metabolism of OO phenols are highly dependent on the individual [125]. At this point, it must be noted that none of the detected metabolic derivatives of secoiridoids are commercially available and consequently cannot be used as standard compounds for comprehensive identification. Consequently, their identification is based on suggested structures and knowledge on the phase I and II metabolic reaction possibly occurring in human system.

Under the same framework of investigation, the bioavailability of OBs has been explored in human urine [138]. Authors identified 60 metabolites, with Oleu aglycone and Lig aglycone derivatives being the most abundant. Authors noticed that absorption and metabolism seem to differ greatly between the various categories of polyphenols and the most abundant metabolites derive from phenolic compounds containing a catechol group, such as HTyr and Oleu [138]. Based on results from previous investigations on the metabolism of Oleu and Lig aglycones and the polar HTyr and Tyr, absorption was measured in eight healthy ileostomy subjects, as was urinary excretion in the ileostomy subjects and in 12 volunteers with a colon [134]. Subjects consumed three different supplements containing 100 mg of OO phenols on separate days in random order. Ileostomy subjects consumed a supplement with mainly nonpolar phenols, one with mainly polar phenols and one with the parent compound, Oleu. Subjects with a colon consumed a supplement without phenols (placebo) instead of the supplement with Oleu. Ileostomy effluent and urine were collected for 24 h after supplement intake. Tyr and HTyr concentrations were low (<4 mol/100 mol of intake) in the ileostomy effluent, and no aglycones were detected. The authors found that absorption of administered Oleu aglycone and HTyr was 55–60% in human subjects. They also underlined that transformation of Oleu and Lig aglycones into HTyr or Tyr is the major reaction in the metabolism of these OO phenolics. This hypothesis was further supported by finding that 15% of Oleu supplement administered to healthy human subjects was excreted in urine as HTyr [134]. Additionally, as in the case of phenylalcohols, secoiridoids are absorbed by the small intestine and their levels are increased dose-dependently in plasma and urine [134]. Studies have shown that they remain highly stable in the mouth but suffer significant losses in the gastric, duodenal and colonic regions, with a recovery rate at the duodenal level ranging between 7% and 34% [4]. Glycosylation and cleavage of glycosidic linkages take part in the secoiridoids absorption, and it is thought that some of them, such as Olea, are absorbed in the small intestine via passive diffusion through the membrane of intestinal cells [90].

Overall, results from in vivo animal studies could be considered in accordance with human interventions. HTyr and Oleu are the most studied compounds, and indirect outcomes exist for the rest of secoiridoids. Unfortunately, absorption rate and extent have not yet been determined for any of the compounds because so far, there is not an appropriate methodology for their detection and quantitation in biological systems. The detected metabolites were the same as in the animal studies, while sulfation and glucuronidation were identified as the major metabolic reactions. It is interesting that HTy-sulf was identified as the most suitable biomarker for monitoring HTyr, Oleu and OO consumption. Oleu was identified as a better supplementation source of HTyr, possibly due to its more resistant chemical structures in human body conditions and its slowest biotransformation to its metabolic derivatives. Few data were found for Olea and Oleo and more research is required to fill the literature gap regarding secoiridoids’ ADME properties.

## 4. Conclusions

Currently, the bioavailability of food bioactives is studied as an integral part to the demonstration of a health benefit. Olive products, rich in OBs, are among the most studied foodstuffs in terms of their chemical composition and health beneficial effects, whereas limited and scattered data exist for their bioavailability. In the current review, the authors attempted to gather all the recent information regarding the bioavailability and metabolism of OBs. The effort targeted the most studied chemical groups of compounds regarding their pharmacological properties, especially phenylalcohols and secoiridoids, namely HTyr, Tyr, Oleo, Olea and Oleu. The aim of the current approach was to highlight the basic metabolic differences of OBs according to their chemical structure. Special attention was also given on human gut microbiome metabolism, which currently prevails in contemporary research design. Based on the first scrutiny of the used literature, the majority of publications concentrate on HTyr, followed by Oleu, due to their commercial availability. As expected HTyr, phenolic extracts and enriched OO expose plentiful data in humans and less in vitro and in vivo due to the ease of their administration. However, this fact is not usually found in the study of natural products’ ADME properties. Our investigation revealed that regarding phenylalcohols, there is strong evidence for ADME(T) properties and there is a large amount of information regarding the bioavailability and metabolism of these compounds. Both HTyr and Tyr have been found to be absorbed in a dose-dependent manner in humans. A variation on results given for HTyr and Tyr is linked with the quantitation methodologies and their endogenous biosynthesis. At least 16 metabolic derivatives have been identified so far, while excretion levels T_max_ and C_max_ have been determined. Bioavailability is the only parameter under question due to analytical challenges existing for their detection in biological matrices.

On the other hand, fewer information was found for secoiridoids. The peculiar and sensitive chemical structure of secoiridoids hinders the design of metabolism studies due to isolation and detection difficulties of these compounds. The bioavailability of Olea and Oleo has been scarcely studied either in in vitro studies or in preclinical and clinical trials. Most research in this field regarding secoiridoids has been focused on Oleu and its aglycone forms. Oleu showed most of the data and witness possible biotransformations of the rest secoiridoids, yet all studies are focused on the phenylalcohol pathway, totally neglecting the elenolic part of the secoiridoids. Additionally, the lack of standard compounds has limited human intervention and is usually investigated as an extract or enriched OO. Generally, it can be presumed that in vitro outcomes can be used as first observations for the design and performance of in vivo experimentations, while the consequent human studies could give the go/no-go decision for the use of such compounds as nutraceuticals. However, more research is required to draw conclusions about the bioavailability and metabolism of such sensitive and unstable compounds. Additionally, this could provide insight regarding extra virgin OO as a more complex food mixture with multiple beneficial health effects. Regarding OBs’ colonic metabolism, limited studies were found and preliminary results around OBs’ metabolism by human gut microbiota could inspire the design of future experimental approaches. Hence, the metabolism studies of OO and OBs specifically is a contemporary subject of research that could contribute significantly to the general understanding of dietary interventions to prevent or even cure human malfunctions, guide personalized nutrition, inform toxicology risk assessment and improve drug discovery and development.

## Figures and Tables

**Table 1:** Representation of the inclusion and exclusion criteria used for the study of the literature.

**Inclusion Criteria**	
**Language**	English and Spanish
**Sample size**	No limit
**Date of publication**	2010–2020 Earlier works related to the topic
**Type of publication:**	Research articles, reviews, short communications
**Exclusion criteria**	
**Language**	Other than English and Spanish
**Type of publication**	Conference abstracts, review meta-analyses, case reports, ecological studies, letters to editors, comments referring to published papers

**Table 2:** Published literature on metabolism of olive bioactive constituents through human studies.

Source of Olive Metabolites (Concentration) [Reference]	Detected or Measured Metabolites in Plasma	Detected or Measured Metabolites in Urine	Detected or Measured Metabolites in Feces	Results and Comments
OO (3.2 HTyr mg) and yogurt (20 mg Htyr)/orally [111]	(Not analyzed)	Htyr, HVAlc,	(Not analyzed)	Htyr excretion was much higher after its administration as a natural component of OO than after its addition to refined OO or yogurt.
Extra virgin OO, refined OO, flax oil, grapeseed oil, margarine, pineapple juice (5 mg of Htyr) [122]	(Not analyzed)	Htyr, Htyr acetate, Tyr, DOPAC, HVAlc	(Not analyzed)	The intake of extra virgin OO, as well as fortified refined olive, flax, and grapeseed oils provided significantly higher urinary contents in Htyr compared with basal urine, whereas Htyr metabolites showed no significant changes. No differences were found between men and women.
30 mL virgin OO (Htyr, Tyr, HVA, *p*-coumaric, Olea, luteolin, pinoresinol, Oleo, acetoxypinoresinol, apigenin) [46]	Tyr-sulf, vanillin-sulf, Htyr-sulf, coumaric acid-sulf, vanillic acid-sulf, HVA-sulf, dihydroferulic acid-sulf, Htyr-glu, apigenin-glu	(Not analyzed)	(Not analyzed)	A μSPE-UPLC-ESI-MS/MS method was developed and applied to determine the phenolic compounds and their glucuronide, sulfate and methylated forms in human plasma after virgin OO ingestion.
OO (20 or 44 or 66 or 84 Htyr μg/mL and 36 or 72 or 110 or 140 Tyr μg/mL) [123]	(Not analyzed)	Htyr, Tyr	(Not analyzed)	Tyr and Htyr are dose-dependently absorbed in humans after ingestion and they are excreted in the urine as glucuronide conjugates.
Virgin OO or extra virgin OO (enriched in secoiridoids-89.4%, phenylalcohols-3.5% and flavonoids-6.0%) [124]	Phenyl alcohols and secoiridoids in sulfated and glucuronated forms	(Not analyzed)	(Not analyzed)	Compared with virgin OO, extra virgin OO increased plasma concentration of the phenol metabolites, particularly HTyr-sulf and vanillin-sulf. After the consumption of virgin OO, the maximum concentration of these peaks was reached at 60 min, while extra virgin OO shifted this maximum to 120 min.
HTyr (5 or 15 mg HTyr) as capsule [25]	(Not analyzed)	Hippuric acid, hydroxypippuric acid, epitestosterone-sulf, 5a-dihydrotestosterone-sulf, HVA, glutamine, HVA-sulf, *p*-cresol-sulf, 1,3-dimethyluric acid, homovanillic aldehyde-sulf.	(Not analyzed)	HTyr supplementation significantly affected the urine metabolome in a dose-dependent manner.
HTyr (2.5 mg/kg BW) as a supplement in an aqueous solution [42]	HTyr, HVAlc	HTyr, HVA, HVAlc, DOPAC in their sulfated and glucuronated form.	(Not analyzed)	The absorption of the HTyr from the supplement was very fast, as the plasma concentration showed maximum levels at 10 min after the administered dose. HTyr was excreted in urine mainly as sulfate and glucuronide conjugates.
50 mL virigin OO (TPC 648 μg/mL, HTyr 70.6 μg/mL and Tyr 27.01 μg/mL) [48]	HTyr-glu, HTyr-sulf, Tyr-glu, Tyr-sulf, HVA-sulf	(Not analyzed)	(Not analyzed)	Quantitative methods were developed for the OO phenolic metabolites in human LDL
40 mL OO (366 mg/kg or 164 mg/kg, or 2.7 mg/kg TPC) [125]	HTyr, Tyr and HVAlc,	(Not analyzed)	(Not analyzed)	Tyr and HTyr were dose-dependently absorbed. Total phenolic compounds in LDL increased in the postprandial state in a direct relationship with the phenolic compounds content of the OO ingested
50 mL extra virgin OO [126]	Tyr and OO phenolics	(Not analyzed)	(Not analyzed)	This methodology allowed the demonstration of the in vitro binding capacity of Tyr
Olive leaf extract capsulated or liquid (Oleu-low dose 51.1 mg Oleu and 9.7 mg HTyr or Oleu-high 76.6 mg Oleu and 14.5 mg HTyr) [127]	HTyr-sulf, HTyr-glu, Oleu	HTyr-sulf, HTyr-glu, Oleu	(Not analyzed)	Gender’s effect on bioavailability, with males displaying greater plasma AUC for conjugated HTyr metabolites. All conjugated HTyr metabolites were recovered in the urine within 8 h. Oleu effectively delivers Oleu and HTyr metabolites to plasma in humans.
25 mL of virgin OO (629 TPC mg/L), refined OO (0.0 TPC mg/L) [128]	HVA-sulf, HTyr-sulf, Tyr-sulf	(Not analyzed)	(Not analyzed)	The phenol concentration of OO modulates the phenolic metabolite content in LDL after sustained, daily consumption. The levels of LDL HTyr-sulf and HVA-sulf, but not of Tyr-sulf, were increased after virgin OO ingestion.
Leaf extract supplemented as capsule or liquid (20 mg Oleu/tablet and 22 mg Oleu/5 mL) [129]	(Not analyzed)	Oleu aglycone-glu, Oleu	(Not analyzed)	Following both chronic and acute ingestion, neither Oleu nor HTyr were detected in urine samples. However, Oleu aglycon-glu was detected in all urine samples up to 6 h following acute ingestion
25 mL virgin OO (49.3 mg/L HTyr)/orally [130]	HTyr, HVAlc and their glucuronated conjugates	HTyr, HVAlc and their glucuronated conjugates	(Not analyzed)	HTyr and HVAlc were quantified in plasma after real-life doses of virgin OO. HTyr absorption was estimated at ∼98% in plasma and urine in conjugated forms, mainly glucuronated conjugates, suggesting extensive first-pass intestinal/hepatic metabolism of the ingested HTyr.
25 mL virgin OO (HTyr 8.5 mg/kg, HTyr acetate 33.4 mg/kg, Olea 269.3 mg/kg, Oleu aglycone 28.5 mg/kg, Tyr 4.4 mg/kg, Oleo 11.4 mg/kg, Lig aglycone 9.9 mg/kg) [52]	(Not analyzed)	(Not analyzed)	HTyr, HTyr acetate, Tyr, phenylacetic acid, 2-(4′- hydroxyphenyl)acetic acid, 2-(3′-hydroxyphenyl)acetic acid, 3-(4′-hydroxyphenyl)-propionic acid	A moderate intake of a phenol-rich OO raised the concentration in human feces of free HTyr and phenylacetic and phenylpropionic acids. The products of colonic catabolism of OO phenolic compounds could be good candidates for novel preventive strategies and in colon and other bowel diseases.
Olive leaf extract (250 mg of olive leaf extract rich in oleuropein (40%) [131]	Luteolin, luteolin-glu, HTyr-sulf-glu, HTyr-glu, HTyr-sulf, HVAlc-glu, Oleu aglycon-glu, Oleu derivative, Oleu-sulf, Tyr-glu, HT acetate glu	HTyr-sulf-glu, HTyr-glu, HTyr-sulf, HVAlc-glu, HVAlc-sulf, Oleu aglycon-glu, Oleu derivative, Oleu-sulf, Tyr-glu, EA, EA-glu, HTyr acetate-glu	(Not analyzed)	Plasma levels of HTyr glu, HTyr-sulf, Oleu aglycon-glu and Oleu aglycon derivative were higher in post-menopausal women. Post-menopausal women excreted less sulfated metabolites in urine than pre-menopausal women.
HTyr 5 or 25 mg Htyr/day HT group supplemented as capsule [132]	(Not analyzed)	Htyr, Htyr-4-glucuronide, Htyr-3-glucuronide, Htyr-3-sulphate, Htyr-4-sulphate	(Not analyzed)	The results show that Htyr given as the foremost component of a nutraceutical preparation is bioavailable and is recovered in the urine chiefly as sulphate-3′, which can be adopted as biomarker of extra virgin OO consumption.
Ethanol (12, 18, 24, 30 and 42 g) or placebo [133]	(Not analyzed)	Htyr, Tyr, DOPAC and HVA	(Not analyzed)	Urinary excretion of Htyr and Tyr increased with ethanol administered dose. This study demonstrates an endogenous production of Htyr and Tyr in relation to ethanol administered dose in humans.
OO phenols (100 mg) as supplements [134]	(Not analyzed)	Htyr, Tyr	(Not analyzed)	Humans absorb a large part of ingested OO phenols and absorbed olive oil phenols are extensively modified in the body.
Olive leaf supplement as capsule or liquid (20 mg Oleu/tablet and 22 mg Oleu/5 mL) [129]	(Not analyzed)	Glucuronic acid conjugates, derived from oleuropein aglycone, uric acid, hippuric acid, 3- and 4-hydroxyhippuric acid	(Not analyzed)	The presence of the metabolites in urine indicates that Oleu reaches systemic circulation and is metabolized in the human system.
Spiked LDL samples with Htyr (0.15 μg/mL) and HVA (1 μg/mL) [135]	Htyr-glu, Htyr-sulf, Tyr-glu, Tyr-sulf, HVA-sulf	(Not analyzed)	(Not analyzed)	A rapid method for the detection and quantification of metabolites of OO phenolic compounds in LDL by SPE and HPLC/ESI-MS/was developed.
50 mL of extra virgin OO (6 mg/20 g Htyr) [96]	EA + H_2_, Olea, Lig aglycone, Lig aglycone + H_2_ + glu, Oleu aglycon + OH, Oleo + H_2_ + glu, apigenin + CH_3_ + glu	Oleu aglycone, Oleu aglycone + H_2_ + glu, methyl-lig aglycone + H_2_ + gluc, lig aglycone +H_2_, Oleu aglycone + OH + sulf, luteolin + CH_3_+ glu, Oleo + glu, Oleu aglycone + OH + glu	(Not analyzed)	Maximum absorption levels of the plasma biomarkers were detected between 0.5 and 2 h, and maximum excretion of urinary biomarkers occurred in the first 4 h after extra virgin OO intake. Plasma secoiridoid compounds were selected as biomarkers to monitor extra virgin OO intake showing good predictive ability according to multivariate analysis.
Virgin OO (80 mg total phenols/kg oil), functional virgin OO enriched with its own phenolics (500 mg total phenols/kg oil), functional virgin OO enriched with its own phenolics plus complementary phenolics from Thyme (500 mg total phenols/kg oil, 50% from olive oil and 50% from thyme respectively) [136]	HTyr-sulf, thymol-sulf, HTyr acetate-sulf, HVAlc-sulf, HVA-sulf, hydroxyphenylpropionic acid-sulf, caffeic acid-sulf	HTyr-sulf, HVAlc-sulf, HTyr acetate-sulf, HVA-sulf, HTyr-glu, HVAlc-glu, hydroxyphenylpropionic acid-sulf, caffeic acid-sulf, thymol-sulf, thymol- glu, *p*-Cymene-diol glu	(Not analyzed)	HTyr-sulf and HTyr acetate-sulf appeared to be suitable biomarkers for monitoring compliance with OO intake as their values in plasma or/and 24 h urine were significantly higher after functional virgin OO compared to baseline pre-intervention concentrations.
25 mL virgin OO (2.7 mg TPC/kg OO or 366 mg TPC/kg OO) [137]	HTyr-sulf, HVA-sulf, HVA-glu	(Not analyzed)	(Not analyzed)	The levels of the studied metabolites increased significantly after high TPC virgin OO ingestion compared to low TPC OO. Virgin OO consumption increases the levels of phenolic metabolites in HDL and thus provides human HDL with more efficient antioxidant protection.
50 mL extra virgin OO (HTyr 8.31 mg/kg, Tyr 5.33 mg/kg, pinoresinol 3.25 m/kg, luteolin 2.65 mg/kg, apigenin 0.64 mg/kg, EA 34.91 mg/kg, lig aglycone 40.58 mg/kg) [138]	(Not analyzed)	50 metabolites, obtained through phase I and phase II metabolic reactions were tentatively identified.	(Not analyzed)	Ten of these biomarkers and more than 50 metabolites obtained through phase I and phase II biotransformation reactions were tentatively identified. Additionally, kinetic studies were conducted on the metabolites identified as possible biomarkers; for most of the compounds, concentrations were maximum in the first two hours.

OO: olive oil, HTyr: hydroxytyrosol, HVAlc: homovanillic alcohol, DOPAC: dihydroxyphenylacetic acid, Tyr: tyrosol, HVA: homovanillic acid, Olea: oleacein, Oleo: oleocanthal, sulf: sulfate, glu: glucuronide, Oleu: oleuropein.

## Supplementary Materials

The following are available online at https://www.mdpi.com/article/10.3390/nu14183773/s1: Table S1: in vitro assays for ADMET properties of olive bioactive constituents; Table S2: published literature on the metabolism of olive bioactive constituents through animal studies.
