# Supplementary material for: Metabolism and Bioavailability of Olive Bioactive Constituents Based on In Vitro, In Vivo and Human Studies
Source: Nutrients. 2022 Sep 13;14(18):3773. doi: 10.3390/nu14183773 (PMC9504511; doi:10.3390/nu14183773)
Supplement: Supplementary file 1 [file nutrients-14-03773-s001.zip › nutrients-1881897-supplementary.pdf]

nutrients

Type of the Paper (Review)

Metabolism and bioavailability of olive bioactive constituents based on *in vitro*, *in vivo* and human studies.

Theodora Nikou [1,#], Maria Eleni Sakavitsi [1,#], Evangelos Kalampokis[1], Maria Halabalaki [1,*]

[1] Division of Pharmacognosy and Natural Products Chemistry, Department of Pharmacy, National and Kapodistrian University of Athens, Greece; th-nikou@pharm.uoa.gr; mariaelenisakavitsi@gmail.com; kalampokis.vag@gmail.com
* Correspondence: mariahal@pharm.uoa.gr; Tel.: +30 210 7274781
# Denotes equal contribution.

(Supplementary material)

nutrients

MDPI

Table S1: *In vitro assays for ADMET properties of olive bioactive constituents.*

Tested compound [reference]	Model System	Identified or measured metabolites	Results
[14C] Htyr [1]	Caco-2 [1]cell monolayers	Homovanillic alcohol	HTyr transport occurred via a passive diffusion mechanism bidirectionally and in a dose-dependent manner.
Oleu [2]	Isolated rat Intestine	-	Oleu was poorly absorbed from isolated perfused rat intestine.
HTyr, Tyr, HTyr acetate [3]	Hepatoma HepG2 [2] cells	-	Methylated and glucuronidated forms of HTyr were detected at 18 h of incubation, together with methylglucuronidated metabolites. HTyr-acetate was largely converted into free HTyr and subsequently metabolized. Tyr was poorly metabolized, with <10% of the phenol glucuronidated after 18 h.
HTyr, Tyr, Oleu [4]	Caco-2/TC7 [3] cell monolayers	Homovanillic alcohol, glutathionylated HTyr	HTyr and Tyr were transferred across human Caco-2 cell monolayers and rat segments of jejunum and ileum and were subject to phase I/II biotransformation. In contrast, there was no absorption of Oleu in either model. However, Oleu was rapidly degraded by the colonic microflora resulting in the formation of HTyr.
HTyr, Tyr, *p*-coumaric acid, pinoresinol, luteolin [5]	Caco-2/TC7 cell monolayers	Methylated and sulfated metabolites	For HTyr the highest metabolism yield corresponded to the formation of methyl HTyr, ranging from 10.7% to 18.6%, dependent on the incubation period. In addition, small quantities of sulfated and methyl-sulfated conjugates of HTyr were also formed after the 6 and 24 h of incubation. Incubation of Caco-2/TC7 cells with Tyr resulted in slow conjugation; the methyl and sulfate conjugates were only quantifiable after 24 h of incubation showing similar metabolism yields to HTyr.
Oleu aglycone, Olea. [6]	Human Caco-2 cell monolayers and isolated lumen of rat intestine (jejunum and ileum)	HTyr, HVAlc, glucuronated derivatives	Compounds were relatively stable under gastric conditions, only undergoing limited hydrolysis and were transferred across a human Caco-2 cells. The compounds underwent extensive metabolism, most notably a two-electron reduction and glucuronidation during the transfer across both the ileum and jejunum.

HTyr ethers (butyl, propyl, ethyl, methyl) [7]	Differentiated Caco-2/TC7 monolayers	Glucuronated and methylated derivatives	The rate of metabolism increased according to the lipohilicity of the ether derivative (butyl > propyl > ethyl > methyl). HTyr ethers are rapidly absorbed across, and partially metabolized by Caco-2/TC7 cell monolayers.
HTyr, HTyr acetate [8]	Caco-2/TC7 cell monolayers	HVAlc, HTyr acetate, HTyr acetate-glu	The acetylation of HTyr significantly increases its transport across the small intestinal epithelial cell barrier.
Olive oil extract, thyme extract and their combination [9]	Caco-2 and HepG2 cell models	-	The bioaccessibility of HTyr was enhanced when both extracts were digested. After Caco-2 cells exposure, no significant differences were observed in HTyr transport.
HTyr as pure compound and in alprerujo powder [10]	Caco-2/TC7 cell monolayers	-	The presence of foods significantly decreased HTyr bioaccessibility and absorption, while β-cyclodextrin had no effect. The presence of other compounds from alperujo in the intestinal compartment reduced HTyr absorption by Caco-2 cells compared to pure standard.
HTyr-glu, Tyr, caffeic, and p-coumaric acids [11]	*In vitro* digestion model and Caco-2/TC7 cells	Ferulic acid	*β*-cyclodextrin did not change the bioaccessibility of the selected phenols. HTyr-glu and caffeic did not cross Caco-2 cell monolayers. β-cyclodextrin moderately but significantly improved the local absorption of Tyr and *p*-coumaric acid.
HTyr [12]	Coculture model (Caco-2/TC7 and HT29-MTX cell lines)	-	The higher the surfactants' concentration in the system the lower the HTyr concentration that penetrated the constructed epithelium, indicating the involvement of the amphiphiles in the antioxidant's absorption and its entrapment in the mucus layer.
Tyr, HTyr, HTyr acetate, Oleu [13]	*In vitro* colon fermentation of human feacal samples	-	The four individual phenols revealed (i) an increase in phenolic acids, (ii) the stability of HTyr and Tyr and (iii) the high degradation of HTyr acetate and Oleu in a faecal culture medium.
HTyr and Tyr [14]	GIDM-colon	Oxidized, ester, methylated, dehydrogenated, dehydroxylated derivatives of HTyr and Tyr along with their dimer and trimer derivatives.	The catechol group played a key role in the metabolic fate of parent compound HTyr and its metabolites. The ortho–hydroxyl group of HTyr seems to promote autooxidation reactions through the formation of ortho–quinones, which trigger a sequential chain of reactions leading to a variety of metabolites. Tyr metabolites are degraded by the microflora of the colon in a similar manner as HTyr.

[1] Caco-2 cells: model system of the human intestinal epithelium; [2] HepG2 cells: model system of the human liver; [3] TC7 cells: spontaneously differentiating clone derived from the original Caco-2 cell population.
HTyr: hydroxytyrosol, Tyr: tyrosol, Oleu: oleuropein, Olea: oleacein, HVAlc: homovanillic alcohol, HTyr-glu: hydroxytyrosol glucuronide

Table S2. *Published literature on the metabolism of olive bioactive constituents through animal studies.*

Substance of administration (concentration) / Subjects / way of administration [reference].	Identified or measured metabolites in plasma	Identified or measured metabolites in urine	Identified or measured metabolites in faeces	Identified or measured metabolites in tissues	Results and comments
HTyr, HTyr acetate, DOPAC (1 and 5 mg/kg) / Sprague–Dawley rats / orally [15].	HTyr, HTyr acetate DOPAC	HTyr acetate, Tyr and HVAlc	(Not analysed)	(Not analyzed)	Different dosages of HTyr, HTyr acetate, and DOPAC are efficiently absorbed in the gastro-intestinal track and have similar metabolism. Their bioavailability was strongly dependent on the derivative considered, dosage, and gender, while different dosages of HTyr, HTyr acetate, and DOPAC do not provide a linear dose- dependent plasma concentration or excretion in urine.
Oleu (100 mg/kg) dissolved in water / male Sprague–Dawley rats /orally [16].	(Not analyzed)	Oleu aglycone, EA, HTyr	Oleu aglycone, EA, HTyr, HVA	(Not analyzed)	De-glucosylation, hydrolysis, oxygenation and methylation were found to comprise the major metabolic pathway of Oleu in rat gastrointestinal tract and three metabolites were absorbed into the blood circulatory system within 24h after oral administration
Oleu (5 mg/kg dissolved in saline) / Sprague–Dawley rats / intravenously [17].	(Not analyzed)	Oxygenated metabolite of Oleu	(Not analyzed)	(Not analyzed)	Oxygenation was found to be the major metabolic pathway of the Oleu in rat blood circulatory system after intravenous administration.
Oleu (0.33/Kg) or with 1.1 g per kg of extra virgin OO / Wistar rats / orally [18].	Oleu, HTyr, HTyr, HVAlc, HVA and EA.	(Not analyzed)	(Not analyzed)	(Not analyzed)	A LC-ESI MS/MS method was developed and validated for the quantification of Oleu, simultaneously with its main metabolites, HTyr, HVAlc, HVA and EA in rat plasma matrix. After sustained low doses of Oleu or extra virgin OO basal levels of HVAlc were found in the blood stream and HTyr was not detected, which indicates that it was metabolized to

					HVAlc or oxidized as it is a very potent antioxidant.
Secoiridoid extract (5 mg phenol/kg) / male and female Wistar rats / orally [19].	HTyr sulf, HVAlc sulf, EA glu	HTyr, HVA, HTyr-sulf, HVA-sulf, EA-sulf, HTyr-4-glu, HVA-glu, EA-glu, methyl-Oleu aglycone-sulf, Oleu, Oleu aglycon-glu	Hydroxybenzoic acid, huppuric acid, phenylacetic acid	Analysis of stomach, small intestine, caecum, liver and kidney. Identification of HTyr, HTyr-sulf, HVA, HVA-sulf, HVAlc-sulf, EA-sulf, Oleu, HTyr-glu, HTyr-acetate-sulf, hydroxyphenylpropionic acid, hydroxyphenylpropionic acid-sulf, hydroxyphenylpropionic acid-sulf, catechol, hippuric acid.	Compared to HTyr and Oleu aglycon and Oleu showed greater stability during digestion, and, consequently, the bioavailability based on the urine excretion of HTyr metabolites was higher. Oleu, as a glycoside molecule, reached the colon unaltered generating more diverse microbial metabolites.
Oleu (25 mg/Kg) dissolved in sodium chloride / Sprague–Dawley rat/ via tail vein injection [20].	Oleu, HTyr	(Not analyzed)	(Not analyzed)	(Not analyzed)	A direct and sensitive reversed-phase high-performance liquid chromatographic assay with fluorescence detection was developed for simultaneous quantification of both Oleu and HTyr in rat plasma.
HTyr, Oleu or secoiridodis extract (5 mg/kg) / Wistar rats / orally [21].	HTyr, HTyr-sulf, HVAlc-sulf	(Not analyzed)	(Not analyzed)	(Not analyzed)	The brain uptake and accumulation of HTyr and its metabolites (HTyr-sulf, HVAlc -sulf) were observed after 21 days of rat diet supplementation of HTyr in its native form or through Oleu derivatives.
Tyr (100 or 200 mg/kg) as a suspension in polyethylene glycol / male Sprague-Dawley rats / by gavage [22].	Tyr-4-sulf and another one unidentified metabolite	Tyr-4-sulf	(Not analyzed)	Analysis of heart, kidney, spleen, lung, liver, epididymal adipose tissue. Identification of Tyr-sulf	Tyr is absorbed rapidly and excreted via the kidney within 8 h. In particular, sulfation in the liver appears to be the major metabolic pathway of Tyr

Olea (300mg/kg), HTyr (100 mg/Kg),and Oleu (300 mg/Kg)/ Wistar ST rats / oral and intravenous administration [23].	HTyr, HVA, and HVAlc	HTyr, HVA, HVAlc, Oleu	(Not analyzed)	Analysis of bile. Identification of Olea, HTyr, Oleu	Olea was readily absorbed and metabolized to HTyr, HVA, and HVAlc in portal plasma. Olea was not observed in the portal plasma, urine and bile.
Olive mill waste water (1, 5, 10 mg/Kg) / Male Sprague-Dawley rats / gastric gavage [24].	(Not analyzed)	HTyr and HTyr glu	(Not analyzed)	(Not analyzed)	HTyr was dose-dependently absorbed and excreted in the urine mostly as a glucuronide conjugate.
HTyr (14)C-labeled / rats / intravenously [25].	HTyr, HTyr oxidized and, methylated metabolites	HTyr, HTyr oxidized, methylated and sulfoconjugated metabolites	HTyr, HTyr oxidized and methylated metabolites	Analysis of brain, heart, kidney, liver, lung, skeletal muscle, gastrointestinal content. Identification of HTyr, DOPAC, DOPAL, HVA, HVAlc and their sulfated derivatives.	90% of the administered radioactivity is excreted in urine collected up to 5 h after injection and about 5% is detectable in feces and gastrointestinal content.
HTyr (1, 10 and 100 mg/Kg) suspended in refined oil / male and female Spargue-Dawley rats / by intragastric gavage [26].	HTyr, HTyr methylated, glucuronater and sulfated derivatives	N-acetyl-5-S-cysteinyl-hydroxytyrosol	(Not analyzed)	(Not analyzed)	Glucuronidation prevails at low HTyr doses close to human dietary intake, sulfation becomes very marked at higher doses, more relevant in the context of supplemented foods or nutraceuticals. Glutathione conjugates of HTyr were reported to be formed in a dose-dependent manner.
HTyr (20 mg/Kg) dissolved in water / Wistar rats / orally [27].	HTyr	(Not analyzed)	(Not analyzed)	(Not analyzed)	A suitable methodology for pharmacokinetic experiments was designed for the recovery of HTyr from rats plasma
3,4-dihydroxyphenylglycol, HTyr and Tyr (extracted from alperujo) / Rowett	3,4-dihydroxyphenylglycol, HTyr and Tyr	(Not analyzed)	(Not analyzed)	Analysis of liver, kidney, heart, muscle, testes. Identification of 3,4-	A novel and highly sensitive method was developed to determine, simultaneously, the concentration 3,4-dihydroxyphenylglycol, HTyr and Tyr in plasma and tissues.

Hooded Lister rats / orally [28].				dihydroxyphenylglycol, HTyr and Tyr.	
Olive cake (dispenced in water) / Wistar rats/ orally [29].	HTyr-sulf, Tyr-sulf, HTyr-glu, Tyr-glu, Oleu derivative, HVA-sulf, vanillic acid-sulf, EA, hydroxybenzoic acid, hydroxyphenyl acetic acid, luteolin-glu.	(Not analyzed)	(Not analyzed)	Analysis of liver, kidney, testicle, brain, spleen, heart, thymus. Identification of HTyr, HTyr-sulf, HTyr-glu, Tyr-suls, Tyr-glu, Oleu derivative, vanillin-sulf, cumaric acid-sulf, cumaric acid-glu, 4-hydroxy-3-methoxyohenylacetaldehyde, vanillic acid, vanillic acid-sulf, cafeic acid, cafeic acid-sulf, HVA, HVA-sulf, ferulic acid-sulf, hydroxyphenylacetic acid, enterolactone, enterolactone-sulf, enterolactone-glu, luteolin.	After a single ingestion of olive oil phenolic compounds, these were absorbed, metabo- lized and distributed through the blood stream to practically all parts of the body, even across the blood-brain barrier.
HTyr (1, 10 and 100 mg/kg) / rats / orally [30].	HTyr, HTyr-sulf, HTyr-glu, HVA, HVA-sulf, HVA-glu.	(Not analyzed)	(Not analyzed)	Analysis of liver, kidney, heart and brain. Identification of HTyr, HTyr-sulf, HTyr-glu, HVA, HVA-sulf, HVA-glu, HVAlc, HVAlc-glu, HVAlc-sulf.	HTyr is accumulated in a dose-dependent manner not only in urine and plasma, but also in the liver, kidney and brain
HTyr (23.5 mg and 25.5 mg) Tyr (14.7 mg and 14.4 mg)/ Sprague-Dawley Rats/ orally and intravenously [31].	(Not analyzed)	HTyr and Tyr	HTyr and Tyr	(Not analyzed)	Oral bioavailability for HTyr was estimated at 99% when administered in an olive oil solution and 75% in an aqueous. Oral bioavailability was estimated at 98% when Tyr was administered in an olive oil solution and 71% in an aqueous.

Oleo (0.1 mg/mL) in transport medium / Sprague–Dawley rats / Single-pass intestinal perfusion [32].	Oleo, Oleo + OH, Oleo + H_2O, Oleo + H_2 + glu, Oleo + H_2O + glu	(Not analyzed)	(Not analyzed)	Analysis of intestinal lumen samples. Identification of Oleo, Oleo + OH, Oleo + H_2O, Oleo + H_2 + glu, Oleo + H_2O + glu	Oleo has a moderate-to-low oral absorption; Oleo was poorly absorbed in the intestine, as indicated by the low effective permeability coefficient (2.23 ± 3.16 × 10−5 cm/s) and apparent permeability coefficient (4.12 ± 2.33 × 10−6 cm/s).
Oleo (0.3 mg/ mL refined olive oil) / Sprague Dawley rats / orally [33].	Oleo, Tyr, Oleo + H_2, Oleo + OH, Oleo + OH + H_2O, Oleo + H_2O	(Not analyzed)	(Not analyzed)	Analysis of brain, heart, intestine, kidneys, liver, lungs, skin, spleen, stomach, thyroids. Identification of Oleo, Tyr, Oleo + H_2, Oleo + OH, Oleo + OH + H_2O, Oleo + H_2O.	Oleo was only detected in the stomach and intestine samples. Moreover, at 2 and 4.5 h, the concentration in the stomach decreased by 36% and 74%, respectively, and in the intestine by 16% and 33%, respectively. Ten Oleo metabolites arising from phase I and phase II reactions were identified. The metabolites were widely distributed in rat tissues, and the most important metabolizing organs were the small intestine and liver. The two main circulating metabolites were the conjugates Oleo + OH + CH_3 and Oleo + H_2O + glu.
Olea (0.15 mg/mL HBSS) / Sprague-Dawley rats / single-pass intestinal perfusion [34].	Olea, HTyr, Olea + H_2, Olea + OH, Olea + H_2O, Olea + CH_3, Olea + OH + CH_3, Olea + H_2O + CH_3, Olea + H_2 + glu, Olea + H_2O + glu, Olea + H_2O + CH_3 + glu	(Not analyzed)	(Not analyzed)	Analysis of lumen and ileum tissue. Identification of HTyr, Olea + H_2 Olea + OH, Olea + H_2O, Olea + CH_3, Olea + OH + CH_3, Olea + H_2O + CH_3, Olea + H_2 + glu, Olea + H_2O + glu, Olea + H_2O + CH_3 + glu.	Olea was mostly metabolized by phase I reactions, undergoing hydrolysis and oxidation, and metabolite levels were much higher in the plasma than in the lumen. Olea was well absorbed in the intestine, with an intestinal permeability similar to that of the highly permeable model compound naproxen.

HTyr: hydroxytyrosol, DOPAC: dihydroxyphenylacetic acid, Tyr: tyrosol, Oleu: oleuropein, EA: elenolic acid, HVA: homovanillic acid, HVAlc: homovanillic alcohol, OO: olive oil, sulf: sulfate, glu: glucuronide, DOPAL: dihydroxyphenylacetaldehyde.

nutrients MDPI

References

1. Manna, C.; Galletti, P.; Maisto, G.; Cucciolla, V.; D'Angelo, S.; Zappia, V. Transport mechanism and metabolism of olive oil hydroxytyrosol in Caco-2 cells. *FEBS Lett.* **2000**, *470*, 341–344, doi:10.1016/S0014-5793(00)01350-8.
2. Edgecombe, S.C.; Stretch, G.L.; Hayball, P.J. Oleuropein, an antioxidant polyphenol from olive oil, is poorly absorbed from isolated perfused rat intestine. *J. Nutr.* **2000**, *130*, 2996–3002, doi:10.1093/jn/130.12.2996.
3. Mateos, R.; Goya, L.; Bravo, L. Metabolism of the olive oil phenols hydroxytyrosol, tyrosol, and hydroxytyrosyl acetate by human hepatoma HepG2 cells. *J. Agric. Food Chem.* **2005**, *53*, 9897–9905, doi:10.1021/jf051721q.
4. Corona, G.; Tzounis, X.; Dessì, M.A.; Deiana, M.; Debnam, E.S.; Visioli, F.; Spencer, J.P.E. The fate of olive oil polyphenols in the gastrointestinal tract: Implications of gastric and colonic microflora-dependent biotransformation. *Free Radic. Res.* **2006**, *40*, 647–658, doi:10.1080/10715760500373000.
5. Soler, A.; Romero, M.P.; Macià, A.; Saha, S.; Furniss, C.S.M.; Kroon, P.A.; Motilva, M.J. Digestion stability and evaluation of the metabolism and transport of olive oil phenols in the human small-intestinal epithelial Caco-2/TC7 cell line. *Food Chem.* **2010**, *119*, 703–714, doi:10.1016/j.foodchem.2009.07.017.
6. Pinto, J.; Paiva-Martins, F.; Corona, G.; Debnam, E.S.; Jose Oruna-Concha, M.; Vauzour, D.; Gordon, M.H.; Spencer, J.P.E. Absorption and metabolism of olive oil secoiridoids in the small intestine. *Br. J. Nutr.* **2011**, *105*, 1607–1618, doi:10.1017/S000711451000526X.
7. Pereira-Caro, G.; Mateos, R.; Saha, S.; Madrona, A.; Espartero, J.L.; Bravo, L.; Kroon, P.A. Transepithelial transport and metabolism of new lipophilic ether derivatives of hydroxytyrosol by enterocyte-like Caco-2/TC7 cells. *J. Agric. Food Chem.* **2010**, *58*, 11501–11509, doi:10.1021/jf101963b.
8. Mateos, R.; Pereira-Caro, G.; Saha, S.; Cert, R.; Redondo-Horcajo, M.; Bravo, L.; Kroon, P.A. Acetylation of hydroxytyrosol enhances its transport across differentiated Caco-2 cell monolayers. *Food Chem.* **2011**, *125*, 865–872, doi:10.1016/j.foodchem.2010.09.054.
9. Rubió, L.; Macià, A.; Castell-Auví, A.; Pinent, M.; Blay, M.T.; Ardévol, A.; Romero, M.P.; Motilva, M.J. Effect of the co-occurring olive oil and thyme extracts on the phenolic bioaccesibility and bioavailability assessed by in vitro digestion and cell models. *Food Chem.* **2014**, *149*, 277–284, doi:10.1016/j.foodchem.2013.10.075.
10. Malapert, A.; Tomao, V.; Dangles, O.; Reboul, E. Effect of Foods and β-Cyclodextrin on the Bioaccessibility and the Uptake by Caco-2 Cells of Hydroxytyrosol from Either a Pure Standard or Alperujo. *J. Agric. Food Chem.* **2018**, *66*, 4614–4620, doi:10.1021/acs.jafc.8b00556.
11. Malapert, A.; Tomao, V.; Margier, M.; Nowicki, M.; Gleize, B.; Dangles, O.; Reboul, E. B-Cyclodextrin Does Not Alter the Bioaccessibility and the Uptake By Caco-2 Cells of Olive By-Product Phenolic Compounds. *Nutrients* **2018**, *10*, doi:10.3390/nu10111653.
12. Mitsou, E.; Dupin, A.; Sassi, A.H.; Monteil, J.; Sotiroudis, G.T.; Leal-Calderon, F.; Xenakis, A. Hydroxytyrosol encapsulated in biocompatible water-in-oil microemulsions: How the structure affects in vitro absorption. *Colloids Surfaces B Biointerfaces* **2019**, *184*, doi:10.1016/j.colsurfb.2019.110482.
13. Mosele, J.I.; Martín-Peláez, S.; Macià, A.; Farràs, M.; Valls, R.M.; Catalán, Ú.; Motilva, M.J. Faecal microbial metabolism of olive oil phenolic compounds: In vitro and in vivo approaches. *Mol. Nutr. Food Res.* **2014**, *58*, 1809–1819, doi:10.1002/mnfr.201400124.
14. Sakavitsi, M.E.; Breynaert, A.; Nikou, T.; Lauwers, S.; Pieters, L.; Hermans, N.; Halabalaki, M. Availability and Metabolic Fate of Olive Phenolic Alcohols Hydroxytyrosol and Tyrosol in the Human GI Tract Simulated by the In Vitro GIDM–Colon Model. *Metab.* 2022, *12*.
15. Domínguez-Perles, R.; Auñón, D.; Ferreres, F.; Gil-Izquierdo, A. Gender differences in plasma and urine

metabolites from Sprague–Dawley rats after oral administration of normal and high doses of hydroxytyrosol, hydroxytyrosol acetate, and DOPAC. *Eur. J. Nutr.* **2017**, *56*, 215–224, doi:10.1007/s00394-015-1071-2.

16. Lin, P.; Qian, W.; Wang, X.; Cao, L.; Li, S.; Qian, T. The biotransformation of oleuropein in rats. *Biomed. Chromatogr.* **2013**, *27*, 1162–1167, doi:10.1002/bmc.2922.
17. Zhou, T.; Qian, T.; Wang, X.; Li, X. Application of LC - MS / MS method for the in vivo metabolite determination of oleuropein after intravenous administration to rat. **2011**, *2011*, 1360–1363, doi:10.1002/bmc.1609.
18. Bazoti, F.N.; Gikas, E.; Tsarbopoulos, A. Simultaneous quantifi cation of oleuropein and its metabolites in rat plasma by liquid chromatography electrospray ionization tandem mass spectrometry. **2010**, *2009*, 506–515, doi:10.1002/bmc.1319.
19. López de las Hazas, M.C.; Piñol, C.; Macià, A.; Romero, M.P.; Pedret, A.; Solà, R.; Rubió, L.; Motilva, M.J. Differential absorption and metabolism of hydroxytyrosol and its precursors oleuropein and secoiridoids. *J. Funct. Foods* **2016**, *22*, 52–63, doi:10.1016/j.jff.2016.01.030.
20. Tan, H.W.; Tuck, K.L.; Stupans, I.; Hayball, P.J. Simultaneous determination of oleuropein and hydroxytyrosol in rat plasma using liquid chromatography with fluorescence detection. *J. Chromatogr. B Anal. Technol. Biomed. Life Sci.* **2003**, *785*, 187–191, doi:10.1016/S1570-0232(02)00855-3.
21. De, M.L.; Godinho-pereira, J.; Macià, A.; Almeida, A.F. Brain uptake of hydroxytyrosol and its main circulating metabolites : Protective potential in neuronal cells. *J. Funct. Foods* **2018**, *46*, 110–117, doi:10.1016/j.jff.2018.04.028.
22. Lee, D.H.; Kim, Y.J.; Kim, M.J.; Ahn, J.; Ha, T.Y.; Lee, S.H.; Jang, Y.J.; Jung, C.H. Pharmacokinetics of tyrosol metabolites in rats. *Molecules* **2016**, *21*, 128, doi:10.3390/molecules21010128.
23. Kano, S.; Komada, H.; Yonekura, L.; Sato, A.; Nishiwaki, H.; Tamura, H. Absorption, Metabolism, and Excretion by Freely Moving Rats of 3,4-DHPEA-EDA and Related Polyphenols from Olive Fruits (Olea europaea). *J. Nutr. Metab.* **2016**, *2016*, doi:10.1155/2016/9104208.
24. Visioli, F.; Caruso, D.; Plasmati, E.; Patelli, R.; Mulinacci, N.; Romani, A.; Galli, G.; Galli, C. Hydroxytyrosol, as a component of olive mill waste water, is dose- dependently absorbed and increases the antioxidant capacity of rat plasma. *Free Radic. Res.* **2001**, *34*, 301–305.
25. Angelo, S.; Manna, C.; Migliardi, V.; Mazzoni, O.; Morrica, P.; Capasso, G.; Pontoni, G.; Galletti, P.; Zappia, V. Pharmacokinetics and Metabolism of Hydroxytyrosol, a Natural Antioxidant from Olive Oil. *Drug Metab. Dispos.* **2001**, *29*, 1492–1498.
26. Kotronoulas, A.; Pizarro, N.; Serra, A.; Robledo, P.; Joglar, J.; Rubió, L.; Hernaéz, Á.; Tormos, C.; Motilva, M.J.; Fitó, M.; et al. Dose-dependent metabolic disposition of hydroxytyrosol and formation of mercapturates in rats. *Pharmacol. Res.* **2013**, *77*, 47–56, doi:10.1016/j.phrs.2013.09.001.
27. Ruiz-Gutiérrez, V.; Juan, M.E.; Cert, A.; Planas, J.M. Determination of hydroxytyrosol in plasma by HPLC. *Anal. Chem.* **2000**, *72*, 4458–4461, doi:10.1021/ac000121h.
28. Rodríguez-Gutiérrez, G.; Wood, S.; Fernández-Bolaños Guzmán, J.; Duthie, G.G.; de Roos, B. Determination of 3,4-dihydroxyphenylglycol, hydroxytyrosol and tyrosol purified from olive oil by-products with HPLC in animal plasma and tissues. *Food Chem.* **2011**, *126*, 1948–1952, doi:https://doi.org/10.1016/j.foodchem.2010.12.044.
29. Serra, A.; Rubió, L.; Borràs, X.; Macià, A.; Romero, M.P.; Motilva, M.J. Distribution of olive oil phenolic compounds in rat tissues after administration of a phenolic extract from olive cake. *Mol. Nutr. Food Res.* **2012**, *56*, 486–496, doi:10.1002/mnfr.201100436.
30. López de las Hazas, M.C.; Rubió, L.; Kotronoulas, A.; de la Torre, R.; Solà, R.; Motilva, M.J. Dose effect on the uptake and accumulation of hydroxytyrosol and its metabolites in target tissues in rats. *Mol. Nutr. Food Res.* **2015**, *59*, 1395–1399, doi:10.1002/mnfr.201500048.

31. Tuck, K.L.; Freeman, M.P.; Hayball, P.J.; Stretch, G.L.; Stupans, I. The in vivo fate of hydroxytyrosol and tyrosol, antioxidant phenolic constituents of olive oil, after intravenous and oral dosing of labeled compounds to rats. *J. Nutr.* **2001**, *131*, 1993–1996.
32. López-yerena, A.; Vallverdú-queralt, A.; Mols, R.; Augustijns, P.; Lamuela-raventós, R.M.; Escribano-ferrer, E. Absorption and intestinal metabolic profile of oleocanthal in rats. *Pharmaceutics* **2020**, *12*, doi:10.3390/pharmaceutics12020134.
33. López-Yerena, A.; Vallverdú-Queralt, A.; Jáuregui, O.; Garcia-Sala, X.; Lamuela-Raventós, R.M.; Escribano-Ferrer, E. Tissue Distribution of Oleocanthal and Its Metabolites after Oral Ingestion in Rats. *Antioxidants* 2021, *10*.
34. López-Yerena, A.; Pérez, M.; Vallverdú-Queralt, A.; Miliarakis, E.; Lamuela-Raventós, R.M.; Escribano-Ferrer, E. Oleacein Intestinal Permeation and Metabolism in Rats Using an In Situ Perfusion Technique. *Pharmaceutics* **2021**, *13*, 719,doi:http://dx.doi.org/10.3390/pharmaceutics13050719.
